# Comparative genomics reveal pathogenicity‐related loci in *Pseudomonas syringae* pv.* actinidiae* biovar 3

**DOI:** 10.1111/mpp.12803

**Published:** 2019-04-26

**Authors:** Zhibo Zhao, Jiliang Chen, Xiaoning Gao, Di Zhang, Jinlong Zhang, Jing Wen, Huqiang Qin, Ming Guo, Lili Huang

**Affiliations:** ^1^ State Key Laboratory of Crop Stress Biology for Arid Areas; and College of Plant Protection Northwest A&F University Yangling 712100 P. R. China; ^2^ The Key Laboratory of Biotechnology for Medicinal Plants of Jiangsu Province Jiangsu Normal University No. 101 Shanghai Rd Tongshan District Xuzhou 221116 P. R. China

**Keywords:** bacterial canker of kiwifruit, Hfq, population, pathogenicity, T3SS, transcriptional regulation

## Abstract

Bacterial canker of kiwifruit, is a severe global disease caused by *Pseudomonas syringae* pv. *actinidiae* (Psa). Here, we found that Psa biovar 3 (Psa3) was the only biovar consisting of three widely distributed clades in the largest Chinese kiwifruit cultivated area. Comparative genomics between the three clades revealed 13 polymorphic genes, each of which had multiple intra‐clade variations. For instance, we confirmed that the polymorphic *copA* gene, which encodes a periplasmic protein CopA that is translocated by the Twin‐arginine targeting (Tat) system, was involved in copper tolerance. We also found extensive variation in pathogenicity amongst strains within each genetically monomorphic clade. Accordingly, the pathogenic determinants of Psa3 were identified via a genomic comparison of phenotypically different strains within each clade. A case study of the high‐ and low‐virulence strains in the clade 2 of Psa3 revealed that an *hfq* variant involved in *in vitro* growth and virulence, while a conserved locus 930 bp upstream of the *hrpR* gene in the Type III secretion system (T3SS) cluster was required for full pathogenicity on kiwifruit and elicitation of the hypersensitivity response on non‐host *Nicotiana benthamiana*. The ‘‐930’ locus is involved in transcriptional regulation of *hrpR/S* and modulates T3SS function via the hierarchical ‘HrpR/S‐HrpL‐T3SS/effector’ regulatory cascade in Psa. Our results provide insights into the molecular basis underlying the genetic diversification and evolution of pathogenicity in Psa3 since kiwifruit canker emerged in China in the 1980s.

## Introduction

Emerging infectious plant diseases raise much concern due to their negative effects on global food security and indirectly on human health (Morens and Fauci, 2013; Subbarao *et al*., 2015). Canker of kiwifruit pandemic caused by *Pseudomonas syringae* pv. *actinidiae* (Psa) biovar 3 has been recorded in all major kiwifruit cultivated areas since a sudden outbreak in 2008 in central Italy, and is currently seriously threatening global kiwifruit production (Donati *et al*., 2014; Scortichini *et al*., 2012; Vanneste, 2017). There have been at least four other genetically distinct Psa populations (biovar 1, 2, 5 and 6) responsible for outbreaks of canker disease on kiwifruit over the past three decades (1984 to present), but they have had limited geographical distributions (Chapman *et al*., 2012; Ferrante *et al*., 2015; Fujikawa and Sawada, 2016; McCann *et al*., 2017; Sawada *et al*., 2016). In fact, each biovar represented a distinct lineage with its own unique repertoire of accessory genes including effectors and toxins (McCann *et al*., 2013, 2017). However, extensive potential genetic divergences within each of Psa biovars 1, 2 and 3 have been revealed by Multiple Locus Variable number tandem repeat Analysis (MLVA) and genomic analysis in Psa (Ciarroni *et al*., 2015; McCann *et al*., 2017). For instance, the pandemic Psa biovar 3 (Psa3) was composed of several recently evolved clonal complexes in China, of which only one is responsible for the current global canker disease (McCann *et al*., 2017). The formation of each clonal complex may result from the niche‐adapting evolutionary processes.

Generally, biological evolution is driven by genetic variation, including point mutations (single nucleotide polymorphisms [SNPs] and short insertions/deletions [INDELs]), DNA rearrangement (insertion, deletion and duplication caused by recombination), and horizontal gene transfer (HGT) (Arber, 2008; Bartoli *et al*., 2016). Genetic variations responsible for changes in bacterial pathogenicity and ecological fitness have been studied extensively in human pathogens and recently in plant pathogens (Coombes, 2016; Guidot *et al*., 2014; Hottes *et al*., 2013; Sokurenko *et al*., 1999). In many cases, disease outbreaks have been caused by different bacterial genetic lineages established through the acquisition of pathogenic determinants and/or genes conferring increased transmission capacity of the pathogen (Bandyopadhyay and Frederiksen, 1999; Bartoli *et al*., 2016; Klemm *et al*., 2016; Morens and Fauci, 2013). Therefore, the genetic variants amongst different clonal complexes within Psa3 may be associated with the pandemic potential of the pathogen. Interestingly, we have observed extensive variation in pathogenicity in genetically very similar Psa3 strains, driving us to investigate the underlying genetic basis during the short evolutionary process in Psa3 as well. For example, there are only 20 SNPs across the whole genome of three Psa3 strains, which showed different pathogenicity and *in vitro* growth patterns. Presumably, the divergent pathogenicity may be caused by a limited number of genetic variations.

In recent years, whole‐genome sequencing (WGS) has been used to identify the specific genetic changes that have occurred during the course of evolution, and has become a compelling approach to study the fundamental mechanisms and processes that drive evolution (Dettman *et al*., 2012; Didelot *et al*., 2016). For instance, evolutionary genomic methods have been well used in experimental evolution (Dettman *et al*., 2012; Guidot *et al*., 2014), molecular epidemiology (Kamath *et al*., 2016; Köser *et al*., 2012; Roetzer *et al*., 2013) and clinical diagnosis (Reuter *et al*., 2013) of many epidemic pathogens. In *Pseudomonas syringae*, a genomic region associated with woody hosts (WHOP) was revealed by the comparison of the draft genome sequences of strains isolated from woody hosts with those of other *P. syringae* strains that infect herbaceous hosts (Caballo‐Ponce *et al*., 2017). During clonal expansion in Psa3, two low‐virulence strains that did not elicit the hypersensitive response (HR) on tobacco and eggplant leaves were recoded, and their phenotypic changes were caused by the insertion of ISPsy31 and ISPsy36 in the* hrpS* and *hrpR* genes, respectively, as revealed by comparative genomics (Firrao *et al*., 2018). Therefore, genomic comparison of the pandemic pathogen Psa3 at the clonal level could provide clues for pathogenic or fitness determinants.

In this study, we found that three clonal complexes within Psa3 were widely distributed in the Shaanxi Province, which is the largest kiwifruit cultivation area in China. Their distribution, pathogenicity patterns and genomic contexts were investigated to reveal the diversification and short‐time evolution of Psa3. Comparative genomics amongst three clonal complexes revealed adaptive genes in response to stresses during Psa3 evolution. Further, the extensive pathogenic diversity amongst Psa3 strains and within each clonal complex suggested a rapid evolution of pathogenicity in the field. According to the variation in the genome and pathogenicity within each genetically monomorphic clonal complex of Psa3, we proposed using comparative genomics to identify the pathogenic or fitness determinants of Psa. Here, we provide a case study of comparative genomics between high‐ and low‐virulence strains in a clonal complex of Psa3. We recovered an *hfq* variant involved in *in vitro* growth and virulence, while a conserved locus 930 bp upstream of the *hrpR* gene in the Type III secretion system (T3SS) cluster was required for full pathogenicity and HR elicitation activity. Our results provide insights into the molecular basis underlying the genetic diversification and pathogenicity evolution of Psa3 since the emergence of canker in kiwifruit in China in the 1980s.

## Results

### Three lineages within Psa3 responsible for kiwifruit canker disease in Shaanxi Province, China

Since 2010, we have isolated 106 Psa strains (Table S1) from trunks, canes, leaves, buds and flowers of *Actinidia chinensis* var. *chinensis* and var.* deliciosa* in five representative micro‐regions throughout Shaanxi Province, which accounts for half of the Chinese kiwifruit cultivation and production. All of the strains were Polymerase Chain Reaction (PCR) positive for Psa‐specific primers KNF/KNR (Koh and Nou, 2002), PsaF/R (Balestra *et al*., 2013) and P0F/P6R (Gallelli *et al*., 2014). Nineteen strains from different sources, of which five have been characterized as Psa3 by rep‐PCR finger printing (Zhao *et al*., 2013), were selected for multilocus sequence analysis (MLSA) based on the concatenated housekeeping gene sequences (*gyrB*‐*rpoD*‐*pgi*‐*acnB*‐*cts*) (Tables 1 and S2). All of them, together with the Psa strains from Italy and New Zealand, belong to Psa3 (Fig. S1).

**Table 1:** Strains from Shaanxi Province, China used for the pathogenicity assay and multilocus sequence analysis (MLSA).

No.	Strain	Host^a^	Isolation date (year, month)	Pathogenicity assay	Rep‐PCR^b^	MLSA	Clade
1	M6(CH2010‐6)	‘Hongyang’	2010, 06	Y			1
2	M7	‘Hongyang’	2010, 06	Y		Y	1
3	M228(C9)	‘Hongyang’	2010, 12	Y		Y	2
4	M401	‘Hort16A’	2012, 05	Y		Y	2
5	M227	‘Qinmei’	2010, 12	Y	Y	Y	2
6	M256	‘Hayward’	2011, 03	Y	Y	Y	3
7	JX1321	‘Hayward’	2010, 10	Y	Y		2
8	M254‐3	‘Hayward’	2011, 03	Y		Y	3
9	M301‐4	‘Hayward’	2011, 03	Y		Y	3
10	M377‐1	‘Hayward’	2012, 03	Y		Y	3
11	M378‐1	‘Hayward’	2012, 03	Y			3
12	M111	‘Xuxiang’	2010, 10	Y	Y	Y	3
13	M336‐2	‘Xuxiang’	2011, 06	Y			3
14	M336‐3	‘Xuxiang’	2011, 06	Y	Y	Y	3
15	M334‐2	var. *deliciosa*	2011, 06	Y		Y	3
16	M339‐1	var. *deliciosa*	2011, 06	Y			1
17	M122	‘Hongyang’	2010, 06	Y	Y		1
18	M23	‘Hongyang’	2010, 06	Y	Y		1
19	M242‐1	‘Hongyang’	2011, 02	Y			2
20	M272‐3	‘Hongyang’	2011, 03	Y		Y	1
21	M350‐1	‘Hongyang’	2011, 10	Y		Y	2
22	M350‐3	‘Hongyang’	2011, 10	Y		Y	2
23	M353‐1	‘Hongyang’	2011, 10	Y		Y	1
24	M531	‘Hongyang’	2013, 03	Y		Y	2
25	M333‐1	‘Hort16A’	2011, 06	Y			2
26	M333‐3	‘Hort16A’	2011, 06	Y	Y		2
27	M402	‘Hort16A’	2012, 05	Y		Y	2
28	M218	‘Xixuan’	2010, 12	Y	Y	Y	2
29	M342‐1	‘Xixuan’	2011, 10	Y		Y	2
30	M303‐3	‘Huayou’	2011, 03	Y	Y		2

a Cultivars of *Actinidiae chinensis* var. *chinensis*: ‘Hongyang’, ‘Hort16A’ and ‘Xixuan’; cultivars of *A. chinensis* var.* deliciosa*: ‘Hayward’, ‘Xuxiang’ and ‘Qinmei’; cultivar ‘Huayou’ is a cross of var.* deliciosa *and var.* chinensis*.

b ‘Y’ in the Rep‐PCR column indicates the strains used by Zhao *et al*. (2013).

**Figure 1:**
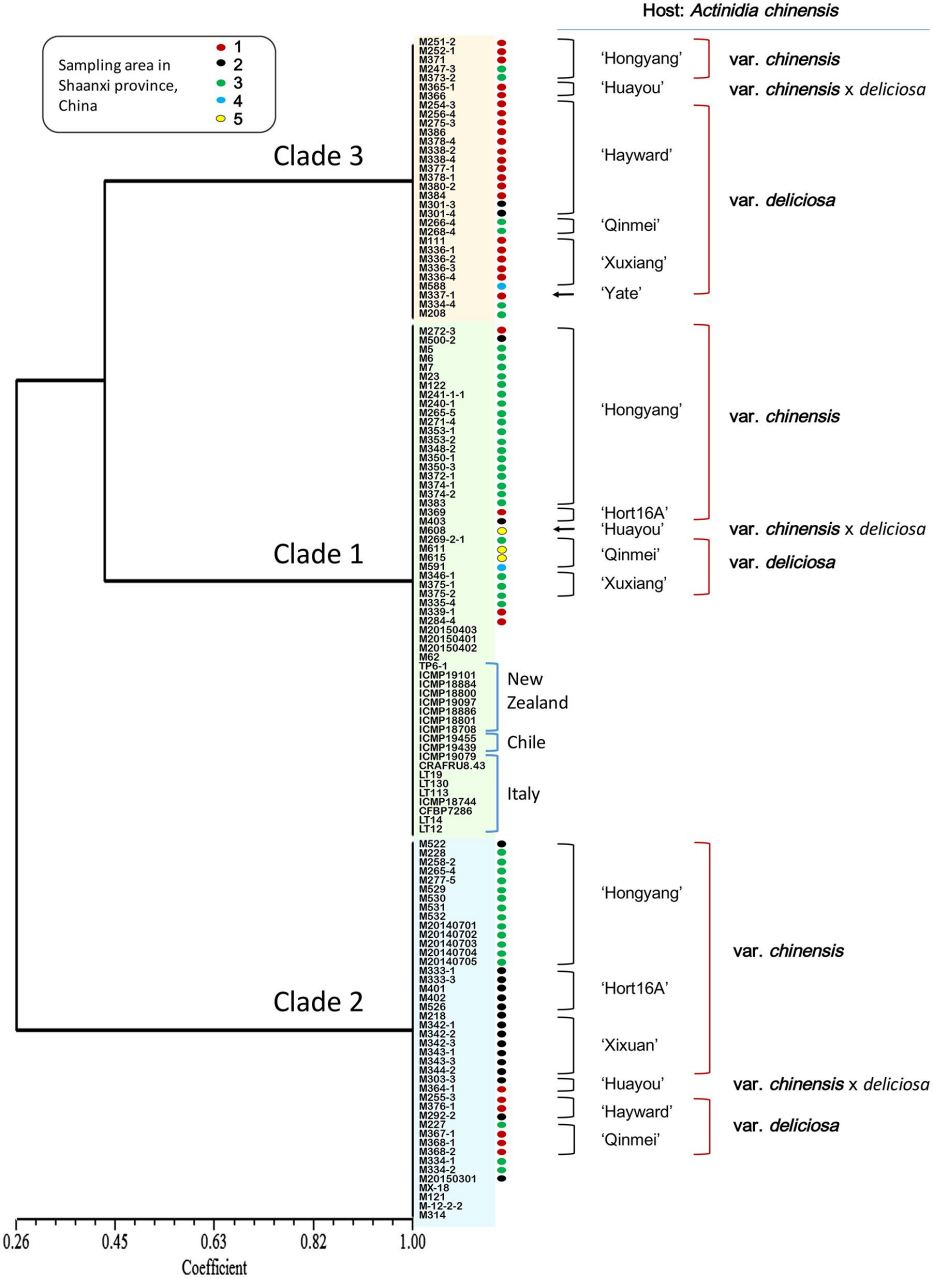
Three clades within *Pseudomonas syringae* pv. *actinidiae* biovar 3 (Psa3) that are widely distributed in Shaanxi Province, China. All 106 Psa strains from Shaanxi Province, China, together with the global biovar 3 strains, were clustered into three clades using seven accessory genetic loci.

**Figure 2:**
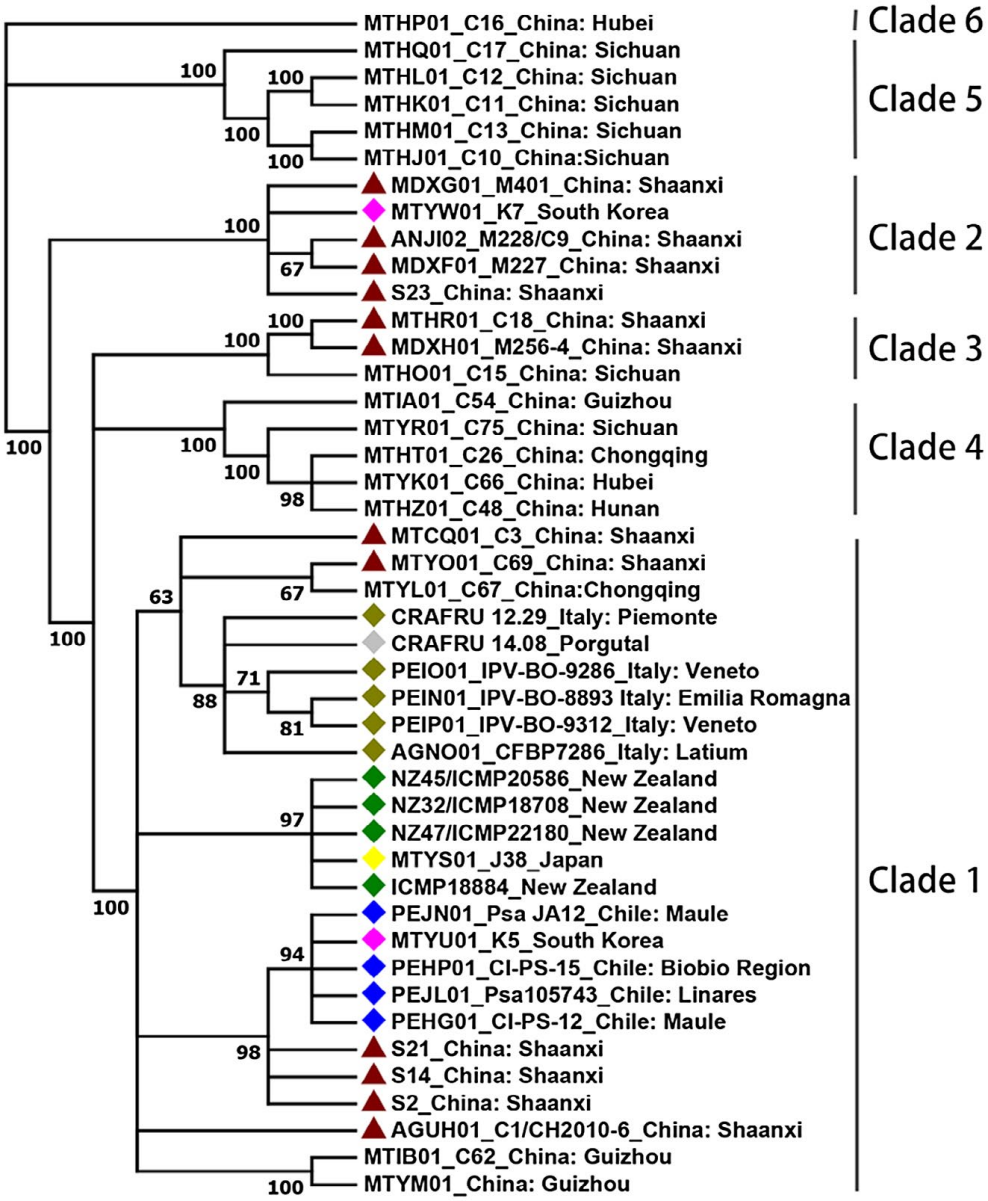
Maximum likelihood tree of 44 representative *Pseudomonas syringae* pv. *actinidiae* biovar 3 (Psa3) strains from different countries based on genomic data. The tree was built based on the bulk of single nucleotide polymorphisms (SNPs) (excluding SNPs due to recombination) from genomic data of the strains shown in Table S3 using REALPHY (defaults, ICMP 18884) and PhyML 3.1 with the GTR model (1000 bootstraps). The red solid triangle indicates Psa3 strains from Shaanxi Province, China. The diamond indicates Psa3 strains isolated outside of China. At least six clades within Psa3 were found across China, while the global Psa3 strains mainly clustered in clade 1 with nine Chinese strains.

**Figure 3:**
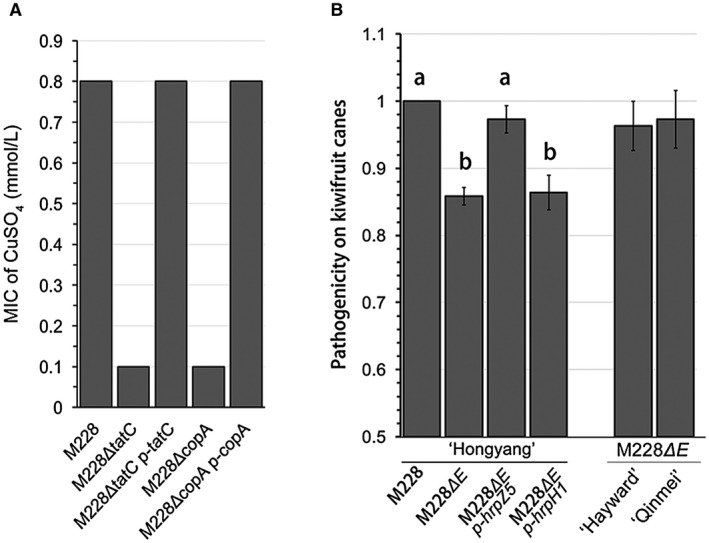
The polymorphic genes* copA* and *hopZ5* revealed by comparative genomics amongst three clades within *Pseudomonas syringae* pv. *actinidiae* biovar 3 (Psa3) are required for copper resistance and pathogenicity, respectively. (A) The minimal inhibitory concentration (MIC) of CuSO_4_ for the *tatC* and *copA* mutants of Psa3 determined by the agar‐dilution method. The LB plates contained eight twofold serial dilutions of CuSO_4_ with a starting concentration of 3.2 mM. An *in‐frame* deletion of either the *copA* or *tatC* gene resulted in increased susceptibility to CuSO_4_, while the changed phenotype could be fully complemented by expressing the corresponding genes. (B) HopZ5 was required for the full virulence of Psa3 during infection. An *in‐frame* deletion of the *hopZ5‐hopH1* cluster from M228 resulted in M228*ΔE*, which showed significantly reduced pathogenicity on canes of *A. chinensis* var. *chinensis *‘Hongyang’, but not on canes of *A. chinensis* var. *deliciosa *‘Hayward’ and ‘Qinmei’. The reduced pathogenicity could be restored by expressing *hopZ5* (M228*ΔE p‐hrpZ5*), but not *hopH1* (M228*ΔE p‐hrpH1*). The lesion length measured at 15 days post‐inoculation (dpi) of each mutant was normalized to that of M228. The experiments were repeated three times with similar results.

However, MLVA and genomic analysis suggested that Psa3 in China may be composed of several recently evolved clonal complexes (Ciarroni *et al*., 2015; McCann *et al*., 2013, 2017). To uncover the genetic differences within Psa3, we clustered the 106 Psa strains into three clades using seven loci including Type III effector (T3E) genes and the putative Type VI substrate genes (Fig. 1, Table S2). Clade 1 consisted of 35 Shaanxi strains plus the global biovar 3 strains whose genome sequences are available in the National Centre for Biotechnology Information (NCBI) database. Clade 2 consisted of 40 Shaanxi strains including strain M228 (WGS accession: ANJI02), which has been shown to be divergent from the global Psa3 strains (McCann *et al*., 2013). Clade 3 was comprised of 31 Shaanxi Psa3 strains. To exclude the bias from gene selection in the clustering method, we attempted to construct a clustering tree using genome‐wide polymorphism data. Genome data of five Psa3 strains from Shaanxi have been deposited in NCBI. We sequenced seven additional Shaanxi Psa strains (150X coverage) in this study. A clustering tree (Fig. 2) was built based on the bulk of SNPs (excluding SNPs due to recombination) from genome data of 44 Psa3 strains, and showed a similar topology to that presented in Fig. 2. This similarity indicates that the aforementioned seven loci are sufficient to reliably distinguish strains of each clade within Psa3. However, Psa3 strains from clade 1 have also been isolated in other Chinese provinces (Guizhou Province and Chongqing Province), New Zealand, Italy, Portugal, Chile, Japan and South Korea; and clades 2 and 3 have also been found in South Korea and in the Sichuan Province in China, respectively (Fig. 2). Moreover, several additional Psa3 lineages have been described in China, including a widely distributed clade found in Guizhou, Sichuan, Chongqing, Hunan and Hubei Provinces (Fig. 3, McCann *et al*., 2017).

In Shaanxi Province, the three clades were widely spread before 2010 because they accounted approximately the same percentage of isolates during each year of 2011 and 2012. They could also be found on kiwifruit trees of all common cultivars in all sampling regions (Fig. 1). We conducted principal component analysis and stepwise discriminant analysis to determine the factors (host variety and host tissue) that may affect the distribution patterns of the three clades. There was no preferential selection of Psa3 on type of kiwifruit tissues, but the population distribution correlated significantly with host variety. Specifically, clade 3 strains were more frequently isolated on *A. chinensis* var.* deliciosa* trees, while clades 1 and 2 made up most strains found on *A. chinensis* var.* chinensis* (Fig. 1). The niche preference might be linked to their genetic context established in the evolutionary process encountering different stresses.

### Genetic causes underlying Psa3 diversification

To determine the genetic nature underlying the evolution of the three Psa3 clades, we detected divergent mutations and their adaptive effects based on WGS data using a computational pipeline. In addition to the most divergent integrative and conjugative element (ICE) (Colombi *et al*., 2017; Mazzaglia *et al*., 2012, McCann *et al*., 2013), the small variants (SNPs and INDELs) and genes subjected to gain or loss events were detected. The core genome of Psa included 3916 single‐copy orthologues shared by four biovars, Psa1, 2, 3 and 5. Across the core genome, variants were detected in only 216 orthologues amongst three Psa3 clades. In the accessory genome, 76 genes were affected by small variants amongst the three Psa3 clades, and 363 genes were present or absent specifically in a certain clade likely resulting from horizontal‐transfer mediated gains or losses of genes.

Although the majority of the affected genes in both the core and accessory genome were defined as clade‐specific, there were 13 polymorphic genes, each of which had multiple intra‐clade variations (Table 2). For instance, we identified the polymorphic *copA* gene encoding a periplasmic multi‐copper oxidase (CopA) with the capability of transforming the toxic Cu(I) to Cu(II). The CopA protein was predicted to be a substrate of the Tat system (Twin‐arginine translocation system) using the TatFIND (Dilks *et al*., 2003) and TatP (Bendtsen *et al*., 2005) tools. We showed that both the *tatC* mutant, which was defective in Tat system function, and the *copA* mutant exhibited increased susceptibility to copper (Fig. 3A). The wild‐type Psa strain M228 had a MIC (minimal inhibitory concentration) of 0.8 mM CuSO_4_ determined by an agar‐dilution method, while the *tatC* and *copA* mutants displayed approximately eightfold copper susceptibility, with a MIC of 0.1 mM CuSO_4_ (Fig. 3A). This indicated that CopA confers copper tolerance in Psa3. Compared to *copA*_clade1_, a nonsynonymous SNP ‘G1196A’ and a nonsynonymous SNP ‘T746G’ were detected in *copA*_clade2_ and *copA*_clade3_, respectively. Although the deletion of *copA* in clade 2 strain M228 can be complemented by each of the three *copA* variants, the *copA*_clade1_ seems to be more efficient with a MIC of 1 mM CuSO_4_ (Fig. S3A), which in accordance with the observations for copper tolerance of the field strains (Fig. S3B). The mutation sites in *copA* from clade 2 and clade 3 apparently have negative effects on copper tolerance. Similarly, a natural 6‐aa deletion of CopA resulted in susceptibility to copper of *Xanthomonas oryzae* pv. *oryzae* (Kong *et al*., 2018). However, acquisition of copper resistance via HGT of the ICE‐ or plasmid‐borne *copABCD* operon has been reported in Psa (Colombi *et al*., 2017; Masami *et al.*, 2004). The ICE‐ or plasmid‐borne *copA* in* copABCD* operon showed over 70% similarity at the amino acid level to the chromosomal *copA* gene in Psa and could restore the copper resistance of M228Δ*copA* at different levels (Fig. S3A,C). Thus, it would be of great interest to investigate the role of the SNPs in* copA*. However, formation of the *copA* variants in Psa is probably not a response to the copper sprays in field.

**Table 2:** Hyper‐mutagenic genes that harbour multiple variants amongst three Psa3 clonal complexes.

No.	Affected genes^a^	Length (aa)	Products	Core / accessory^b^
1	IYO_RS03030	118	Hypothetical protein	Core
2	IYO_RS10350	200	Glutathione S‐transferase	Core
3	IYO_RS05600	272	Peptidase M48	Core
4	IYO_RS00670	437	FAD‐dependent oxidoreductase	Core
5	IYO_RS15660	440	Glycosyl transferase	Core
6	IYO_RS13735	506	ABC transporter substrate‐binding protein	Core
7	IYO_RS12640	554	Cyclic peptide transporter	Core
8	IYO_RS08460	592	Copper resistance protein A	Core
9	IYO_RS15145	337	Peptide ABC transporter ATP‐binding protein	Accessory
10	IYO_RS20685	342	Hypothetical protein, downstream of Major Facilitator Superfamily (MFS) transporter	Accessory
11	IYO_RS13525	598	Type VI secretion system protein ImpG	Accessory
12	IYO_RS15440	1039	Acriflavine resistance protein B	Accessory
13	IYO_RS12175	6088	Filamentous hemagglutinin	Accessory

a Genetic loci are designated according to the genome information of ICMP 18884 (NZ_CP011972).

b Core indicates genes shared by all Psa biovars, whereas ‘accessory’ indicates genes shared by Psa3, but that may not be present in other Psa biovars.

### Pathogenicity changes partially related to Psa3 diversification

**Figure 4:**
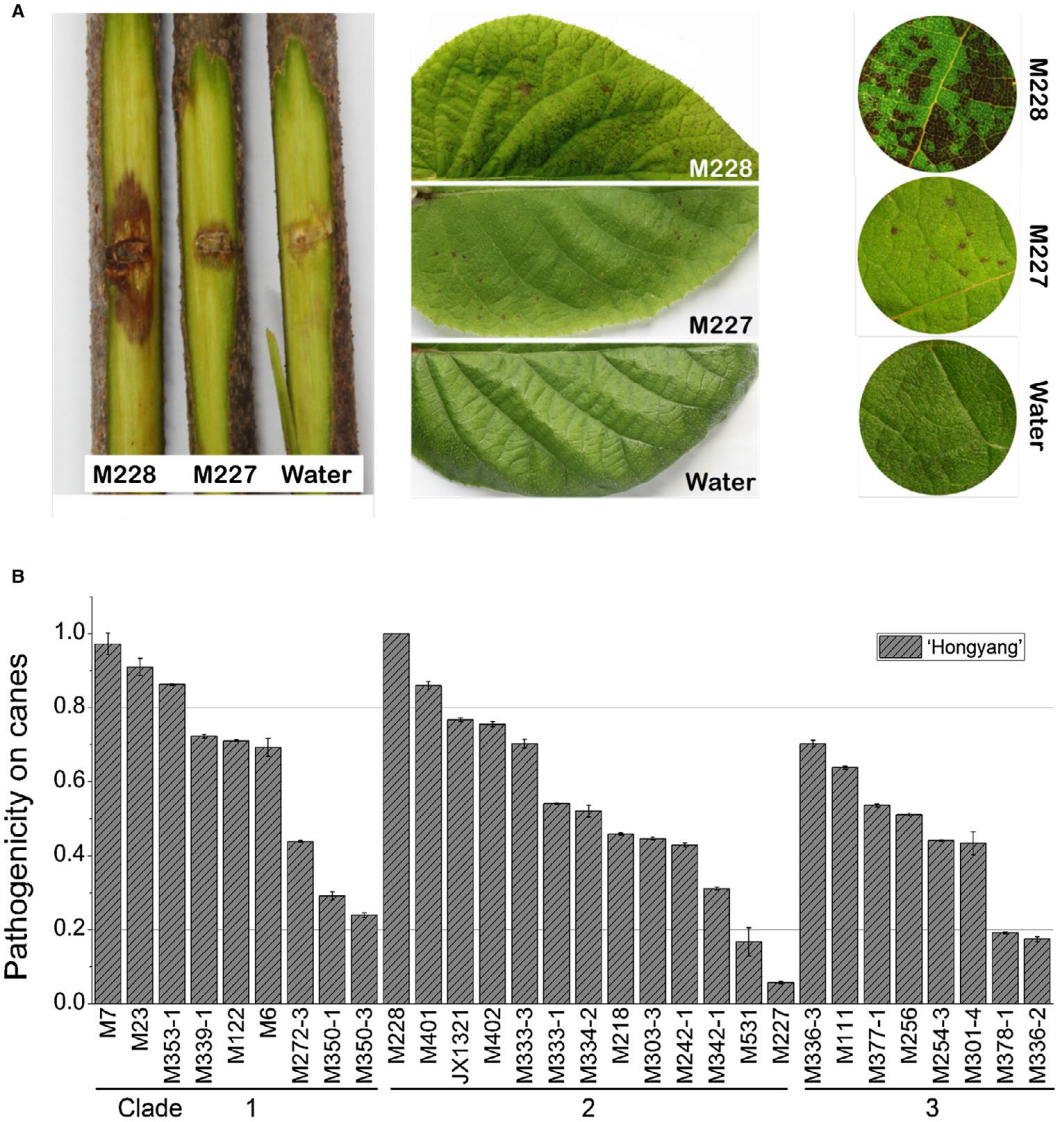
Reliable indoor bioassay methods to test the pathogenicity of *Pseudomonas syringae* pv. *actinidiae* (Psa) strains. (A) Three indoor bioassay methods were developed: 1) wound inoculation on detached dormant kiwifruit canes with 10^8^ CFU/mL bacteria, 2) spraying the inoculate on unwounded kiwifruit leaves with 10^6^ CFU/mL bacteria, 3) vacuum infiltration inoculation on leaf discs with 10^4^ CFU/mL bacteria. The three methods showed very similar results when used to test the pathogenicity of the high‐virulent Psa strain M228 and a low‐virulent Psa strain M227. (B) Diverse pathogenicity of the Psa biovar 3 (Psa3) strains on kiwifruit in Shaanxi Province, China. The pathogenicity patterns of 30 Psa3 strains from Shaanxi Province were obtained by wound inoculation on detached dormant woody canes of *Actinidia chinensis* var.* chinensis* ‘Hongyang’. A variation in pathogenicity was observed amongst strains within each genetically monomorphic clade. The lesion length measured at 15 day post‐inoculation (dpi) of each strain was normalized to that of Psa3 strain M228. The experiments were repeated three times with similar results.

If pathogenicity changes are related to population diversification, the clade‐specific genetic variants are probably pathogenic genes. To investigate the pathogenicity of Psa strains, we developed three laboratory bioassay methods. The three methods showed very similar results when used to test the pathogenicity of the high‐virulence Psa strain M228 and the low‐virulence Psa strain M227 (Fig. 4A). The reliability of the wound inoculation method was further evaluated by inoculating M228 on nine kiwifruit cultivars. The results indicated that *A. chinensis* var.* chinensis* was more susceptible than *A. chinensis* var.* deliciosa* overall and that *A. chinensis* var.* chinensis* ‘Hongyang’ was the most susceptible compared to the rest of the cultivars (Fig. S2). Remarkably, the patterns in disease resistance of the nine cultivars were largely consistent with those observed in kiwifruit orchards (Qin *et al*., 2013), suggesting that the indoor bioassay was robust for evaluating kiwifruit resistance against Psa. We therefore examined the pathogenicity of 30 Psa3 strains on *A. chinensis* var.* chinensis* ‘Hongyang’ using the wound inoculation method. The results showed no significant differences in mean pathogenicity amongst the three Psa3 clades, and the pathogenicity patterns of Psa3 strains were not related to their sources (host variety, geographical origin or infected kiwifruit tissues). However, clade 3 appeared to be less virulent than the other clades, as very high‐virulence strains were not found in this population. Moreover, clade 3 was more frequently isolated from *A. chinensis* var.* deliciosa* trees, clade‐three genetic changes involved in host‐microbe interactions and woody host adaption might be important for this preference.

T3Es, which are expected to mutate frequently enabling pathogens to avoid detection within extant hosts or adapt to new hosts (Karasov *et al*., 2014), were conserved amongst the three clades. However, six T3E genes exhibited SNPs or INDELs across three clades. *HopZ5* was the only T3E gene in clade 3 that was distinct from other Psa3 clades by a nonsynonymous SNP (amino acid sequence of HopZ5_clade3_: T259A). Previously, HopZ5 was found only in strains that are pathogens of woody hosts (Nowell *et al*., 2016), and, in Psa, HopZ5 is unique to the global outbreak Psa3, but is absent in other Psa biovars. Here we confirmed that HopZ5 was related to the virulence of Psa during infection (Fig. 3B). The *in‐frame* deletion of the *hopZ5‐hopH1* cluster from M228 resulted in significantly reduced pathogenicity on canes of *A. chinensis* var. *chinensis* ‘Hongyang’, but not on canes of *A. chinensis* var. *deliciosa* ‘Hayward’ and ‘Qinmei’ (Fig. 3B). Further, the reduced pathogenicity could be restored by expressing *hopZ5*, but not *hopH1* (Fig. 3B). Recent studies showed that HopZ5_Psa3_ localized to the cell periphery and triggered HR‐like cell death (HCD) associated with the plant immunity‐related gene *SGT1* in *Nicotiana benthamiana* (Choi *et al*., 2017), also triggered a strong HR in the *Arabidopsis thaliana* Catania‐1 (Ct‐1) accession but not in Col‐0 and Wassilewskija‐2 (Ws‐2) (Jayaraman *et al*., 2017). Moreover, a suppressor of HopZ5‐triggered immunity in *Arabidopsis*, SOBER1, was identified (Choi *et al*., 2018). However, the roles of the HopZ5_Psa3_ in host‐pathogen interaction and the ‘T259A’ mutation on host preference remain to be determined. We found that the ‘T259A’ mutation of HopZ5 did not affect the HCD induction and subcellular localization in *N. benthamiana* leaves (Fig. S3D,E). We further constructed an *hopZ5*_clade3_ deletion mutant and evaluated its pathogenicity on canes of both ‘Hongyang’ and ‘Hayward’. However, the *hopZ5*_clade3 _showed no difference with *hopZ5*_M228_ in pathogenicity (Fig. S3F). We have tried to identify the host targets of HopZ5 in ‘Hongyang’ and ‘Hayward’ using yeast two‐hybrid assay, but no positive clones have been obtained.

### Pathogenicity variation linked to limited genetic changes within a Psa3 clade

**Figure 5:**
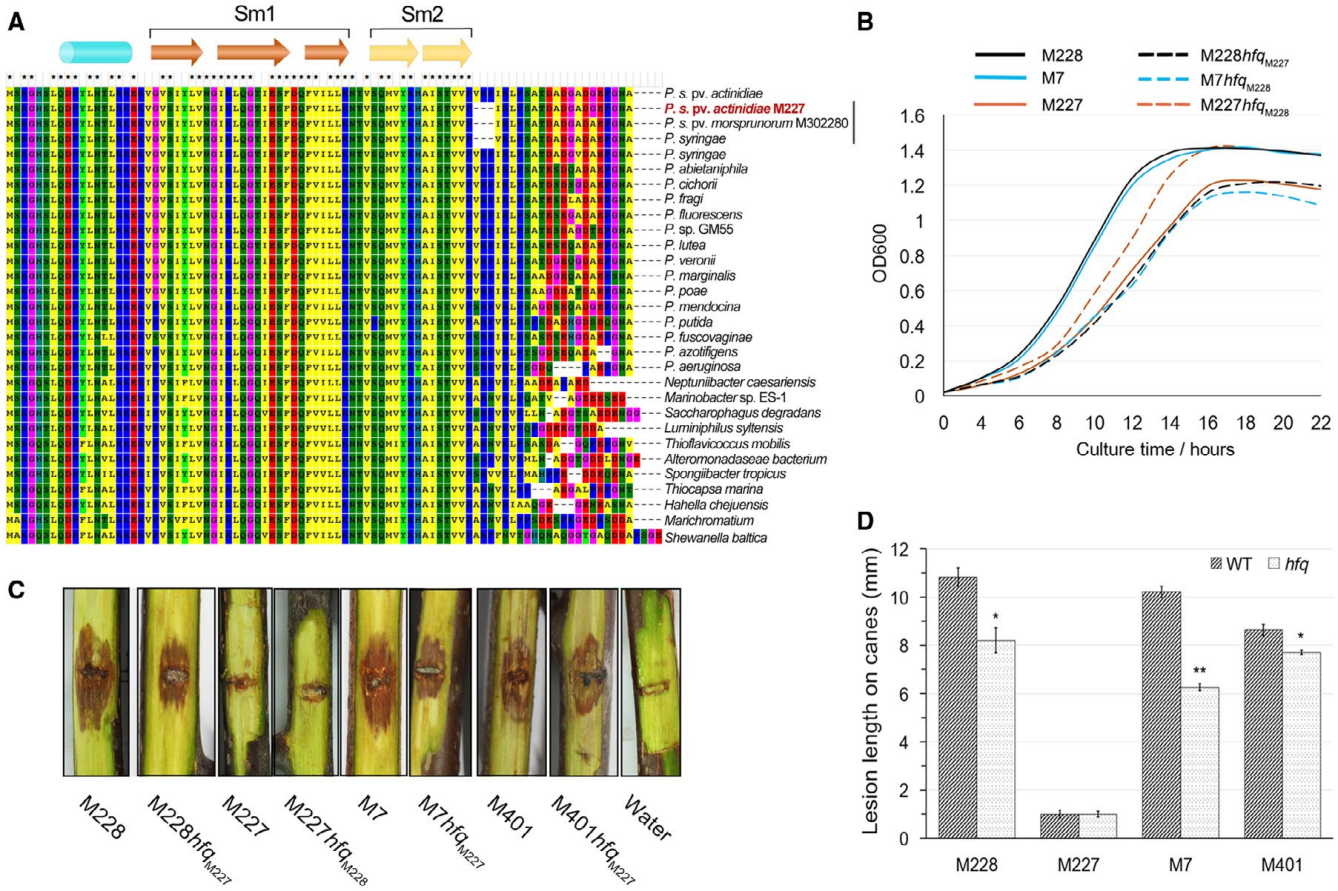
An *hfq* variant is responsible for modified growth and pathogenicity of *Pseudomonas syringae* pv. *actinidiae* biovar 3 (Psa3). (A) Alignment of *hfq* alleles from various bacterial species. The secondary structure of Hfq was indicated on the top. (B) The *Hfq*_M227_ allele has an effect on bacterial growth and involved both a prolongation of the lag phase and a very slight decrease in the final culture density. The bacterial density in LB liquid media was measured at OD600 nM at the indicated time points. (C, D) The *Hfq*_M227_ allele had a slight effect on Psa3 pathogenicity. The *hfq*_M227_ variant caused a slight decrease in the pathogenicity of Psa3 strains M7, M228 and M401, while the *hfq*_M228_ variant did not enhance the pathogenicity of M227. The pathogenicity tests were performed by wound inoculation on detached dormant woody canes of *Actinidia chinensis* var. *chinensis* ‘Hongyang’. Values are means ± standard deviations (SDs). The experiments were repeated twice with similar results.

**Figure 6:**
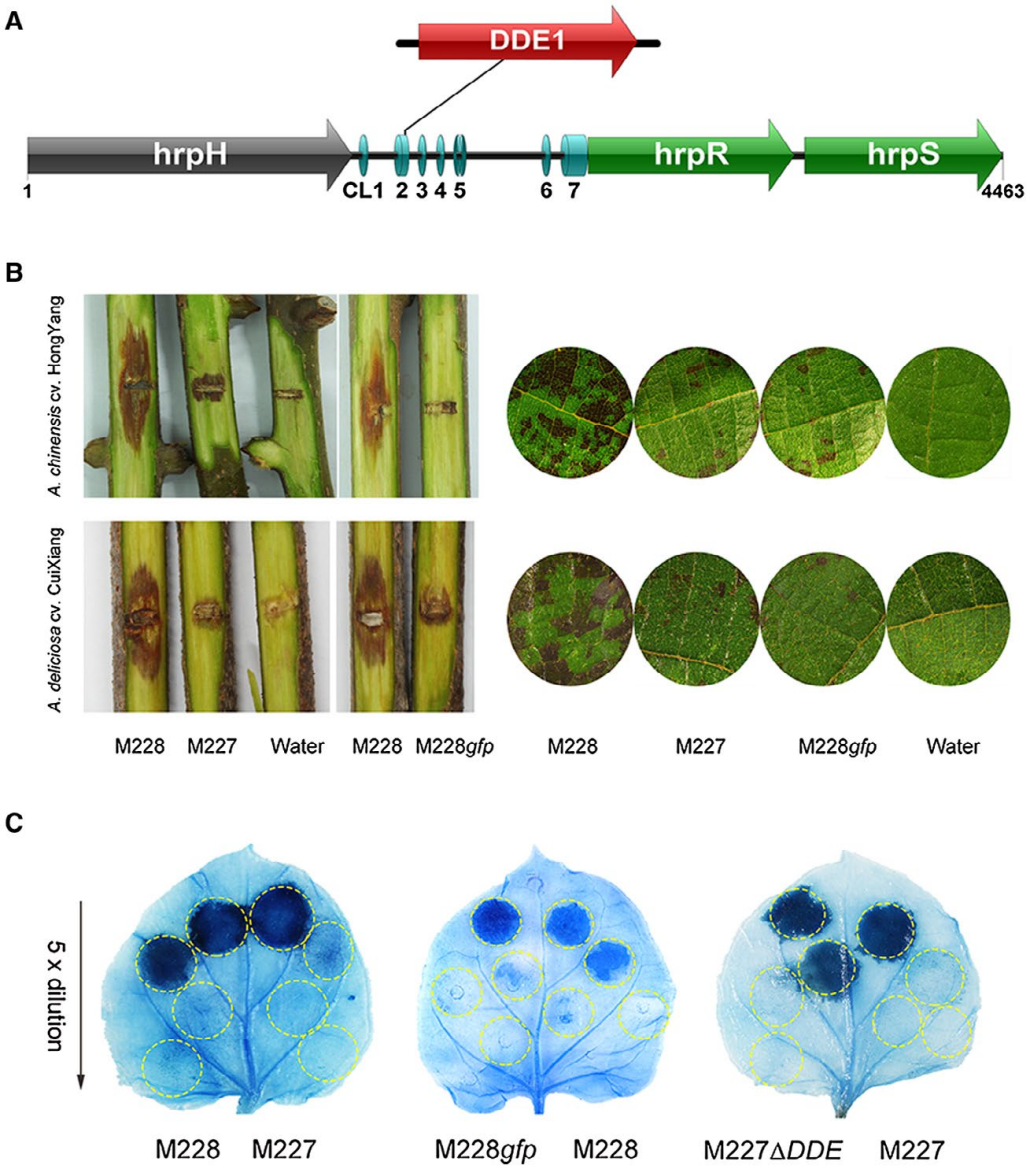
A conserved locus upstream of the *hrpR* gene is required for full pathogenicity and hypersensitive response (HR) elicitation activity in *Pseudomonas syringae* pv. *actinidiae *(Psa). (A) A 1176 bp transposable element is inserted 930 bp upstream of *hrpR/S* in the low‐virulent Psa strain M227. The NCS between* hrpH* and *hrpR *in the Type III secretion system (T3SS) cluster from the *P. syringae* group harbours seven conserved loci, designated CL1 to CL7. The illustration was drawn in IBS 1.03 (Liu et al., 2015) (B) The ‘‐930’ locus is required for pathogenicity in Psa. The up and down bioassay results on *Actinidia chinensis* var *chinensis* ‘Hongyang’ and var* deliciosa *‘Cuixiang’, respectively. Left: Wound inoculation on detached kiwifruit canes at 15 days post‐inoculation (dpi). Right: vacuum infiltration inoculation on leaf discs at 5 dpi. For the inoculation, at least 10 canes or leaf discs were used for each strain. (C) The ‘‐930’ locus is involved in the attenuated capacity to elicit the HR in *Nicotiana benthamina* leaves in M227. Four zones in half a leaf were injected with fivefold diluted bacteria (10^7 ^CFU/mL to 8 x 10^4^ CFU/mL), and three leaves from three independent plants were treated with each strain. M228 is the high‐virulent Psa strain, and in M228*gfp*, an 841 bp *gfp *fragment was artificially inserted at the ‘‐930’ locus. In M227∆*DDE*, the inserted locus was deleted. Experiments were repeated at least twice with similar results.

Remarkably, variations in pathogenicity were observed amongst strains within each genetically monomorphic clade (Fig. 4B), revealing a rapid evolution of pathogenicity. Presumably, the divergent pathogenicity of strains within each genetically monomorphic clade of Psa3 was caused by a limited number of genetic variations. For example, only 20 SNPs were present amongst the three Psa3‐clade 2 strains M228, M227 and M401 (Table S3), which showed diverse phenotypes including pathogenicity and *in vitro* growth. Specifically, compared to the high‐virulence strain M228, M227 showed low‐virulence to both *A. chinensis* var.* chinensis* and *A. chinensis* var.* deliciosa* (Figs 5C and 6B), attenuated capacity to elicit the HR in *N. benthamina* leaves (Fig. 6C), and growth deficiency in both Lysogeny Broth (LB) and minimal liquid media (Fig. 5B). To determine if the changed phenotype was caused by the genetic changes in M227, we investigated the eight loci that are M227‐specific variations found in comparative genomic analyses of the three strains (Table 3). All eight of the variations were validated by PCR and Sanger sequencing using the primers listed in Table S2. The M227‐specific loci included three SNPs and five INDELs and were mainly involved in regulatory processes. We then swapped each of the eight loci between M227 and M228, and evaluated the changes in phenotype. Remarkably, we found that an *hfq* variant was involved in *in vitro* growth and pathogenicity, but was not necessary for pathogenicity, while a conserved locus 930 bp upstream of the *hrpR* gene in the T3SS cluster was required to elicit full pathogenicity and HR.

**Table 3:** M227‐specific variations resulting from comparative genomic analyses of the three strains belonging to Psa3 clade 2.

No.	Variant type	Nucleotide /sequence in M227	Affected genes^a^	Products	Primers for Sanger sequencing
1	SNP	g → a	IYO_RS06590, P29S	Hypothetical protein	PSA184/185
2	SNP	g → a	IYO_RS21050, M87I	DNA ligase‐associated DEXH box helicase	PSA188/189
3	SNP	a → g	IYO_RS24010, RBS	DNA‐binding response regulator	PSA194/195
4	Deletion	‘cgggcgaac’ absence	IYO_RS25325, 3AA deletion	RNA‐binding protein Hfq	hfq‐orf‐F/R
5	Deletion	‘gcgtcgccgt’ absence	IYO_RS02715, frame shift	DNA‐directed RNA polymerase subunit beta	PSA176/177
6	Insertion	1176‐bp *DDE* locus	At locus 930 bp upstream of* hrpR*	‐	PSA198/199
7	Insertion	gg	Non‐coding sequence within the integrative and conjugative element (ICE)	‐	PSA200/201
8	Insertion	106 bp	IYO_RS24140, truncated	Lytic transglycosylase	PSA202/203

a Genetic loci are designated according to the genome information of ICMP 18884 (NZ_CP011972).

### An *hfq* variant responsible for changed growth and slight pathogenicity of Psa3

A DNA deletion was detected in M227 with respect to the other Psa strains corresponding to 3‐amino acid (^65^VRP^67^) at the coiled‐coil C‐terminus of the RNA‐binding protein Hfq, which is involved in multiple regulatory processes via orchestrating mRNA‐sRNA interactions (Vogel and Luisi, 2011) (Fig. 5A). The three amino acids, especially the R_66_ residue, are conserved in Hfq amongst various bacterial species (Fig. 5A). Interestingly, the same deletion was also found in two other *P. syringae* strains, a non‐pathogenic *P. syringae* pv. *morsprunorum* strain M302280 (gb|EGH07934.1) and another *P. syringae* strain (WP_025168020.1). To determine whether this 3‐amino acid deletion has been fixed in the Psa populations in Shaanxi Province, we analysed the *hfq* loci from 105 strains mentioned above using PCR with the primers PSA198/199. Surprisingly, the deletion was only detected in M227 and not in the other strains.

In many bacteria species, loss of Hfq results in reduced fitness, impaired stress response and diminished virulence (Vogel and Luisi, 2011). Functions of Hfq rely on its homohexameric structure, of which each subunit is composed of an amino (N)‐terminal α‐helix followed by five highly twisted and curved antiparallel β‐strands, terminating in an unstructured carboxy (C)‐terminal region (Vogel and Luisi, 2011). The Hfq ring can contact RNA at four sites: the proximal face with the N‐terminal α‐helices, the distal face on the opposite side (Sm2 motif), the rim at the outer ring and the C‐terminal tail. Different RNAs bind to these sites in various configurations (Updegrove *et al*., 2016). Given that the C‐terminal tail of Hfq is important for the interaction with at least some sRNAs (e.g. RydC), the *hfq*_M227_ variant may be responsible for the changed phenotype of M227. To test this, we swapped the* hfq* genes between M227 and M228. The M228*hfq*_M227_ strain showed deficient growth in both LB and minimal liquid media similar to that of M227, while M227*hfq*_M228_ partially restored its growth capacity compared to that of M228 (Fig. 5B). When inoculated on kiwifruit plants, the *hfq*_M227_ variant caused a slight decrease in pathogenicity, while the *hfq*_M228_ variant could not enhance the pathogenicity of M227 (Fig. 5C,D). A similar phenotype was observed for M401*hfq*_M227 _and the clade 1 strain M7*hfq*_M227_ (Fig. 5B,C,D), suggesting that the 3‐amino acid region is important to Hfq functioning in regulating bacterial growth and virulence.

### A conserved locus upstream of the *hrpR* gene is required for full pathogenicity and HR elicitation activity

**Figure 7:**
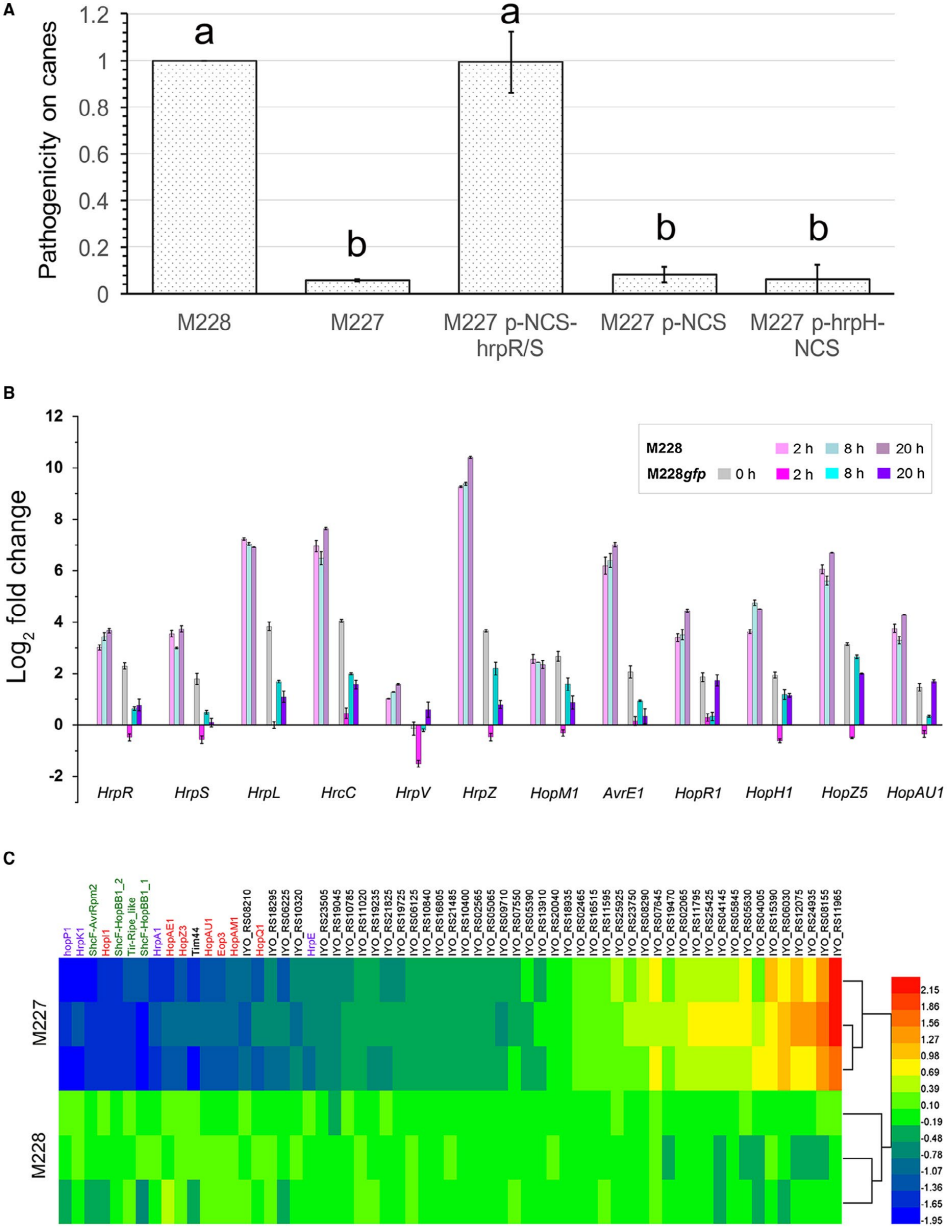
The ‘‐930’ locus upstream of the *hrpR* gene is involved in transcriptional regulation of hrpR/S and its integrity is required for the expression and secretion of Type III secretion system (T3SS) and effectors (T3Es). (1) The pathogenicity‐related ‘‐930’ locus is involved in the transcription of *hrpR/S*, but not *hrpH*. We constructed three mutants of the low‐virulent strain M227 by expressing each of the three fragments, *hrpH*‐NCS, NCS alone and NCS‐*hrpR/S*, cloned from M228. However, only M227 expressing NCS‐*hrpR/S*_M228_ demonstrated significantly increased pathogenicity. (2) The transcriptional patterns of T3SS and effector genes in M228 and the mutant M228*gfp* in the* hrp*‐inducing condition. The nutrient‐rich King’s B (KB) medium and HDM medium were *hrp*‐repressing and *hrp*‐de‐repressing, respectively. Bacteria were cultured in KB at the designated ‘0 h’ and subsequently transferred into HDM medium at a final concentration of 0.02 OD_600_. Quantitative Real‐Time Polymerase Chain Reaction (qRT‐PCR) was performed in a Bio‐Rad IQ5 thermal cycler using SYBR Green reagent. Three replicates were performed for each sample. The results shown are the mean and standard deviation, and the relative expression ratios were compared amongst samples for each gene (Duncan’s multiple range test, *P* < 0.05). Experiments were repeated at least twice with similar results. (3) M227 secreted less Type III pilus, translocator, harpins and effectors in *hrp*‐inducing conditions. The values in the heat‐map are log_2 _(protein_ratio_in_M227/ protein_ ratio_in_M228). The purple, green and red fonts indicate the T3SS structure or helper proteins, chaperons and effectors, respectively. M228 is the high‐virulent Psa strain, and in M228*gfp* an 841 bp *gfp *fragment was artificially inserted at the ‘‐930’ locus.

As M227*hfq*_M228_ did not significantly enhance pathogenicity, other variants were inferred to be involved in Psa pathogenicity. We performed comparative secretome analysis of M227 and M228 using a mass‐spectrometry‐based quantitative method after growing in HDM (*hrp*‐depressing medium) (Stauber *et al*., 2012), which allowed the identification of 61 proteins that were differentially secreted between M227 and M228 (number of unique peptides ≥ 2, *P* < 0.01), of which 40 were down‐regulated and 21 were up‐regulated (Fig. 7C, Table S4). In the set of the most significantly different ( > 1.5‐fold) proteins, 22 and 12 proteins were down‐ and up‐regulated, respectively (Table S4). Remarkably, 15 out of these 61 proteins were found to be T3SS‐associated proteins, and all of them were down‐regulated meeting the 1.5‐fold threshold (Table 4). These proteins include the most differently secreted Type III harpin HopP1, translocator HrpK1 and pilus HrpA1, all of which were the most abundant proteins found in the extracellular fractions of Pto DC3000 after growth in the *hrp*‐inducing medium (Schumacher *et al*., 2014). However, four Type III chaperones ShcF (AvrRpm2), ShcF (HopBB1‐2), Tir (Ripe2‐like) and ShcF (HopBB1‐1), which were identified as differently secreted proteins here, are believed to function as chaperones unfolding and targeting effectors and remaining in the bacterial cytosol (Portaliou *et al*., 2016; Wilharm *et al*., 2007). Further, six T3Es (HopI1, HopAE1, HopZ3, HopAM1, Eop3‐like and hopQ1) were also secreted at lower levels from bacterial cells of M227. Notably, all of the top 16 proteins that were secreted less by M227 were encoded by the HrpL‐mediated genes, which possess a common upstream sequence, the '*hrp* box' (Table 4). This result indicated that M227‐specific loci associated with T3SS expression and secretion are likely to be responsible for the altered phenotype in M227.

**Table 4:** List of secreted proteins whose abundance ratios were reduced by over 1.5‐fold in M227 compared to M228.

No.	Locus^a^	Gene/protein	Hrp_box	M228 / M227
1	IYO_RS14375	HopP1, harpin	+	3.29
2	IYO_RS06905	HrpK1, translocator	+	3.02
3	IYO_RS29250	ShcF‐AvrRpm2, chaperon	+	2.91
4	IYO_RS05155	HopI1, effector	+	2.88
5	IYO_RS03660	ShcF‐HopBB1_2, chaperon	+	2.87
6	IYO_RS09185	Tir‐Ripe2_like, chaperon	+	2.84
7	IYO_RS03720	ShcF‐HopBB1_1, chaperon	+	2.74
8	IYO_RS06785	HrpA1, needle filament protein	+	2.70
9	IYO_RS12205	HopAE1, effector	+	2.67
10	IYO_RS29005	HopZ3, effector	+	2.60
11	IYO_RS02055	Tim44	+	2.56
12	IYO_RS29755	HopAU1, effector	+	2.40
13	IYO_RS13130	Eop3, effector	+	2.35
14	IYO_RS08375	HopAM1, effector	+	2.27
15	IYO_RS08210	Amidinotransferase	+	2.21
16	IYO_RS03520	HopQ1, effector	+	2.20
17	IYO_RS18295	Hypothetical protein	−	1.76
18	IYO_RS06225	Glycine cleavage system protein H	−	1.76
19	IYO_RS10320	Orotidine 5'‐phosphate decarboxylase	−	1.75
20	IYO_RS06810	HrpE, T3SS stator	+	1.71
21	IYO_RS23505	Hypothetical protein	−	1.66
22	IYO_RS19045	2‐pyrone‐4,6‐dicarboxylate hydrolase	−	1.52

a Loci are designated according to the genome information of ICMP 18884 (NZ_CP011972). Loci highlighted in light‐blue are associated with Type III secretion system (T3SS) and effectors (T3Es).

We then deleted the naturally inserted sequence (1176 bp in length, encoding transposable element endonuclease DDE1) upstream of the *hrpR* gene in M227 (Figs 6A and S4). The resulting strain M227∆*DDE* exhibited a significant increase of pathogenicity on kiwifruit plants. We also inserted an 841 bp *gfp* gene derived from the plasmid pDSK‐GFPuv (Wang *et al*., 2007) in M228 at the same locus, resulting in the mutant strain M228*gfp*. We similarly found that the mutant strain M228*gfp* was significantly attenuated in pathogenicity equivalent to that of M227 (Fig. 6B). Meanwhile, HR elicitation in *N. benthamiana* leaves by either M227 or M228*gfp* required at least fivefold more bacterial cells than that of M227*ΔDDE* or M228 (Fig. 6C). These results suggest that this locus is required for Psa pathogenicity. However, the insertion at this locus was not present in the other 105 strains including several low‐virulence strains, suggesting that it was not fixed in Psa populations, likely because the disruption of the locus appeared to not be beneficial for Psa.

We then investigated the underlying mechanism of the locus involved in interactions between pathogen and host/non‐host. The locus located in one of the seven conserved regions of the non‐coding sequence (NCS) between *hrpH* and *hrpR* in the *Pseudomonas* T3SS cluster (Fig. S4), suggesting an important role of the locus in T3SS functioning. However, the roles of the NCS between *hrpH* and *hrpR* are largely unknown. We constructed three mutants of M227 by expressing each of the three fragments, *hrpH*‐NCS, NCS alone and NCS‐*hrpR/S* cloned from M228, respectively. However, only M227 expressing NCS‐*hrpR/S*_M228_ showed increased pathogenicity (Fig. 7A), indicating that this locus likely affects pathogenicity via directly regulating *hrpR/S*.

During infection, HrpR and HrpS, a co‐dependent pair of AAA + bacterial enhancer‐binding proteins (bEBPs), transcriptionally activate the extracytoplasmic function (ECF) subfamily σ factor HrpL, which serves as the master regulator to control the expression of the T3SS and effector genes by binding to a consensus sequence known as the *hrp* box (Bush and Dixon, 2012; Jovanovic *et al*., 2011; Tang *et al*., 2006; Xiao *et al*., 1994). We therefore used quantitative Real‐Time (qRT)‐PCR to perform an overall investigation of the transcriptional expression patterns of T3SS and effector genes in M228 and M228*gfp*, cultured in the *hrp*‐repressing condition (King’s B [KB] medium) and subsequently transferred into the *hrp*‐inducing conditions (HDM medium or *in planta*). The T3SS‐associated genes detected encode bEBPs HrpR and HrpS, regulator HrpL, secretin HrcC, C‐ring protein HrcQa, ATPase HrcN, translocator HrpK1 and harpin HrpZ; and the effector genes detected encode the core effectors AvrE1, HopM1 and HopI1, and the variable/interchangeable effectors HopR1, HopF2, HopAZ1, HopAE1, HopS2, HopY1, HopN1, HopH1, HopQ1, AvrRpm1, HopZ3, HopZ5, AvrPto5, HopD1, double‐copy HopAM1 and the plasmid‐born HopAU1. In the *hrp*‐repressing condition KB medium, M228*gfp* exhibited elevated expression levels of both T3SS and T3E genes compared to those in M228 (Figs 7B and S5A). In the *hrp*‐inducing conditions (both in HDM medium and *in planta*), however, M228*gfp* displayed decreased expression of both T3SS and T3E genes at the early phase similar to those of M228 cultured in KB, and subsequently slightly elevated expression that was even lower than that of itself cultured in KB (Figs 7B and S5). Expression patterns of the T3SS and T3E genes in M228*gfp* in both conditions were different from those in the T3SS deficient mutant M228Δ*hrcS*, which exhibited similar expression patterns compared to the wild type (Fig. S5 A). Notably, given the presence of the HrpR/S‐HrpL‐T3SS/T3Es hierarchical regulatory cascade in *P. syringae*, the modified expression patterns of T3SS/T3E in M228*gfp* were positively attributed to the changes in levels of *hrpR/S* mRNA, which were well correlated with the *hrpL* mRNA here.

Together, these data suggest that the reduced pathogenicity on kiwifruit plants and the attenuated HR elicitation in *N. benthamiana* leaves by M227 might be caused by the decreased expression and secretion of T3SS/T3Es, which is likely to be affected by the disruption of the pathogenicity‐associated locus upstream of the *hrpR* gene. However, we have not determined the molecular nature of the locus regulating *hrpR/S* transcription. In *P. syringae* pv. *tomato* DC3000, several regulators, such as HrpA (Wei *et al*., 2000), GacA/S (Chatterjee *et al*., 2003), the two‐component system RhpR/S (Deng *et al*., 2014; Xiao *et al*., 2007) and the Ca^2+^‐induced two‐component system CvsS/R (Fishman *et al*., 2018), may affect the transcriptional expression of *hrpR/S*. HrpA and GacA, which exhibit high levels of RNA in *hrp*‐inducing medium, likely act as positive regulators upstream of HrpR and HrpS via yet unknown mechanisms in DC3000 (Chatterjee *et al*., 2003; Wei *et al*., 2000). CvsR is the first direct transcriptional activator of *hrpR/S* identified in *P. syringe* (Fishman *et al*., 2018), and has four putative binding sites in the NCS upstream of *hrpR/S* (Fig. S4). In the RhpR/S system, the highly phosphorylated RhpR (RhpR‐P) directly binds to the upstream non‐coding region of *hrpR/S* to repress transcription in *hrp*‐inhibiting conditions, whereas the RhpR‐P is dephosphorylated and present at a low level in *hrp*‐inducing conditions (Deng *et al*., 2014; Xiao *et al*., 2007). It is plausible that RhpR‐P in Psa may bind to the conserved DNA sequence (TGTAA[G/C/A]N6[C/A]TTACA) immediately upstream of the ‘‐930’ locus in CL2 (Fig. S4), which would explain the evaluated transcription of *hrpR/S* in M228*gfp* under *hrp*‐inducing conditions, but not the causes of the reduced transcription of *hrpR/S* when M228*gfp* is subsequently transferred under the *hrp*‐inducing conditions. Moreover, a sequence containing only the immediate upstream putative promoter (the conserved CL7 region shown in Fig S3) and the* hrpR/S* open reading frames (ORFs) is sufficient for *hrpR/S* expression in DC3000 under* hrp*‐inducing medium (Hutcheson *et al*., 2001; Xiao *et al*., 1994). Thus, an unknown negative regulator associated with the CL2 sequence may be present in Psa. An interesting avenue for further research will be the identification and delineation of the unknown negative regulator.

## Discussion

In this study, we investigated the population structure and the genetic causes underlying Psa3 diversification in China’s largest kiwifruit cultivated area. The pathogenicity and distribution patterns of the three Psa3 clades in Shaanxi Province, China extended our understanding of their pandemic potential. We did not observe any differences in pandemic potential between the global pandemic clade 1 and the current endemic clade 2. In fact, clade 2 has been found in South Korea as well (McCann *et al*., 2017). We have tried to determine the genetic basis underlying the evolution of the three clades based on the WGS data. In addition to the ICEs and clade‐specific variants (SNPs and INDELs), we found 13 intra‐clade polymorphic genes, such as the copper‐associated gene *copA* and the virulence‐related T3E gene *hopZ5*. These genes are good candidates for investigating the mechanisms of stress responses in Psa. More importantly, our finding suggested that Psa3 has displayed a rapid evolution in pathogenicity in the field. We found extensive variation in pathogenicity amongst Psa3 strains, and even within each of the three clades. The divergent pathogenicity within each genetically monomorphic clade of Psa3 was likely caused by a limited number of genetic variations. Therefore, genomic comparison of phenotypically different strains within each clade can be used to identify the pathogenic or fitness determinants of Psa. Here, we provide a case study of comparative genomics between high‐virulence strains M228 and M401 and the low‐virulence strain M227, all of which belong to clade 2 of Psa3, revealing two loci involved in bacterial growth, pathogenicity and HR induction. We found an *hfq* variant (the ^65^VRP^67 ^deletion in M227) that was involved in *in vitro* growth and virulence. However, the comparative secretome analysis suggested that the reduced pathogenicity and HR induction of M227 might be caused by the attenuated expression and secretion of T3SS and effector repertoires. The genetic evidence confirmed that a naturally transposable insertion in a conserved locus 930 bp upstream of *hrpR* gene in the T3SS cluster was responsible to the reduced pathogenicity in kiwifruit and HR induction in *N. benthamiana* leaves, which is different from a recent study that two Psa strains, CRAFRU 12.29 and CRAFRU 12.50, did not elicit HR on tobacco and eggplant leaves and were limited in their growth in kiwifruit leaves due to an insertion of ISPsy31 and ISPsy36 in the *hrpS* and *hrpR* genes, respectively (Firrao *et al*., 2018). We further confirmed that the ‘‐930’ locus is involved in transcriptional regulation of *hrpR/S* and modulates T3SS function via the hierarchical ‘HrpR/S‐HrpL‐T3SS/T3Es’ regulatory cascade in Psa. However, we have not yet fully determined the molecular nature of the locus regulating *hrpR/S* transcription, though the presence of an unknown negative regulator associated with the locus is supported. How the phytopathogenic bacteria sense their environment to activate the T3SS remains largely unknown (Chatterjee *et al*., 2003; Deng *et al*., 2014; Fishman *et al*., 2018; Wei *et al*., 2000; Xiao *et al*., 2007). An interesting avenue for further research will be the identification and delineation of this unknown negative regulator in *P. syringae*.

## Experimental Procedures

### Bacterial strains and culture conditions

The bacteria were grown in Luria–Bertani (LB) at 37 °C for *E. coli* strains, 28 °C for *Agrobacterium tumefaciens* strains, and 25 °C for *P. syringae* strains with appropriate antibiotics. The concentrations (mg/L) used with the following antibiotics are: kanamycin, 50; nalidixic acid, 10; and ampicillin, 20. All Psa3 strains (Table S1) were stored in 15% glycerol solution at −80 °C. The MIC of CuSO_4_ for Psa3 was determined by an agar‐dilution method (Wiegand *et al*., 2008). In brief, 1 μL of bacterial suspension (10^8^ CFU/mL) was dotted on the CuSO4‐containing LB agar with a series of dilutions (0.025, 0.05, 0.1, 0.2, 0.3, 0.4, 0.5, 0.6, 0.7, 0.8, 0.9, 1.0, 1.1, .12. 1.3, 1.4, 1.5, 1.6 and 3.2 mmol/L). LB agar without CuSO4 was used as a control. Agar plates were incubated at 25 °C for 48 h.

### MLSA and Psa3 clustering using seven accessory loci

Five housekeeping gene fragments, *acnB*, *gltA*, *gyrB*, *pgi* and *rpoD*, were amplified using the corresponding primer pairs (Table S2), followed by Sanger sequencing. Concatenated sequences of five genes, for a length of 2277 nucleotides, were aligned and used to construct several phylogenetic trees with MEGA6 (version 6.06, Tamura *et al*., 2013) using neighbour‐joining (NJ) with the Kimura 2‐parameter model and maximum likelihood (ML) using the estimated best models. Trees were compared, and the branch robustness was estimated by 1000 bootstrap replicates.

Seven pairs of primers, designed based on seven accessory genes, were used for PCR amplification of 106 Psa3 strains (Table S2). The polymorphic bands were used for the construction of a binary value matrix, representing the absence and presence of bands by 0 and 1, respectively. An NTSYS‐pc version 2.02 programme (Exeter software, Setauket, New York, USA) was used for data analysis using a simple matching coefficient of similarity as a base for dendrogram construction via the UPGMA (unweighted pair group method with arithmetic mean) method (Rohlf, 1998).

### Whole‐genome sequencing and variant discovery

Seven Psa3 strains were selected for genome sequencing. Both the high‐virulence strain M228 and the low‐virulence strain M227 belong to Psa3 clade 2 (designated in this study), whereas the high‐virulence strain M256 belong to Psa3 clade 3. Genomic DNA was extracted using the TIANamp Bacteria DNA Kit (TIANGEN Biotech Co. Ltd., Beijing, China). All genomic sequences were produced with Illumina sequencing using the paired‐end protocol with a read length of 250 nt at the Beijing Novogene Company (Beijing, China). The raw data were filtered using fastq‐mcf (Aronesty, 2013) and quality checked using FastQC (http://www.bioinformatics.babraham.ac.uk /projects/fastqc/). *De novo* assemblies of draft genomes were generated with VAGUE (Powell and Seemann, 2013). The contigs of each assembly were reordered according to the reference complete genome ICMP 18884 (genome, NZ_CP011972; plasmid, NZ_CP011973) using the Mauve Contig Mover (Rissman *et al*., 2009), and gene prediction and annotation of each assembly were performed using the PROKKA pipeline (Seemann, 2014).

For SNP identification and phylogenetic analysis, a full alignment of 21 Psa3 genomes (Table S1) was performed using progressive Mauve (Darling *et al*., 2010; defaults, with iterative refinement and allowing re‐arrangements, ICMP 18884 as reference). The full alignment was parsed to identify genomic regions shared by all strains (locally collinear blocks [LCBs] less than 1 kb were filtered out), resulting in 5 612 915 bp in length. Recombination events in bacterial genomes were detected using ClonalFrame version 1.2 (Didelot and Falush, 2007), and recombination‐related sequences were removed in the subsequent analysis. SNPs were identified, and a SNP tree was constructed based on the ‘clean genomic data’ (shared by all Psa3 strains, recombination‐related sequences removed) using REALPHY (Bertels *et al*., 2014; defaults, ICMP 18884, M228 and M256 as references) and PhyML 3.1 with the GTR model (1000 bootstraps) (Guindon *et al*., 2010).

For identification of the core and accessory genome of Psa, we took a gene‐based approach to define the core genes of Psa. The genomes of Psa1, 2, 3 and 5 strains listed in Table S1 were input into OrthoMCL (Chen *et al*., 2006). A gene was considered core if all Psa genomes had hits with identity ≥ 75% and length ≥ 50 bp. We identified the core genome of Psa1, Psa2, Psa3 and Psa5, including 3915 single‐copy orthologues and the accessory genome including additional 2020 orthologues.

For variant calling and annotation, variant (SNP and INDEL) calling was initially performed for the three newly sequenced strains M227, M401 and M256 using Samtools and GATK package. The INDELs were then blast searched against a custom, local database constructed from all Psa3 genomes, defining the clonal‐specific and strain‐specific INDELs. We used SNPs, which were identified using the ‘clean’ Psa3 genomic data indicated above, to define the clonal‐specific and strain‐specific SNPs. The variant‐affected genes were extracted and annotated using KOBAS 2.0 (statistical test method: hypergeometric test/Fisher's exact test) (Xie *et al*., 2011).

### Pathogenicity tests

We developed three indoor bioassay methods: (1) wound inoculation on detached dormant kiwifruit canes with 10^8^ CFU/mL bacteria, (2) spraying inoculation on unwounded kiwifruit leaves with 10^6^ CFU/mL bacteria, (3) vacuum infiltration inoculation on leaf discs with 10^4^ CFU/mL bacteria. The three methods showed very similar results in all tests. For wound inoculation on detached dormant kiwifruit canes, the Psa‐free healthy dormant kiwifruit woody canes were cut to a length of 50 cm. After surface sterilization with 0.6% sodium hypochlorite for 10 min followed by three washes with sterile water, the woody canes were air dried and were carefully cut with a sterilized scalpel. Also, 10 μL of bacterial suspension at a concentration of 2 × 10^6^ cells/mL was inoculated into each wound area. In all experiments, a replicated mock inoculation with sterile water was included as a control. For each Psa3 strain, at least 20 kiwifruit canes were used for inoculation. The inoculated canes were incubated in a climate chamber with 16 h lighting at 18 ºC and 8 h dark at 14 ºC. The length of lesion was measured 15 days post‐inoculation. At least three independent experiments were carried out.

### Allele replacement and mutant generation

Allele replacements and mutants were constructed using a *SacB*‐based unmarked mutagenesis method previously described (Kvitko and Collmer, 2011). Briefly, for *hfq* allele replacements, an 1860 bp long targeting DNA fragment carrying *hfq* gene was amplified by PCR using primers hfqF/R from either M227 or M228 and cloned into the *BamHI*/*HindIII* sites on the suicide plasmid pK18*mobSacB*. The resulting pK18*mobSacB*::*hfq_M227_* and pK18*mobSacB*::*hfq_M228_* constructs were transformed into *E. coli* strain S17‐1/λ_pir_ and sequenced. The single crossover merodiploid conjugants were selected on LB agar with antibiotics (kanamycin, 50 mg/L; nalidixic acid, 10 mg/L; ampicillin 20 mg/L) and confirmed by PCR to detect the *SacB* gene using primer SacB‐F/R. The PCR positive conjugants were subjected to a counter‐selection on LB agar containing 15% sucrose. The final mutants were confirmed by PCR using the primer pairs hfq‐DF/DR, which should result in a 663 bp product or no product when using Psa *hfq*_M228_ or Psa *hfq*_M227 _as a template, respectively. Four mutants, M228*hfq*_M227_, M7*hfq*_M227_, M401*hfq*_M227_ and M227*hfq*_M228_ were generated and their *hfq* alleles were confirmed by Sanger sequencing.

For deletion of the naturally inserted sequence (encoding endonuclease DDE1) upstream of the *hrpR* gene in the T3SS cluster in M227, a 1470 bp long targeting DNA fragment carrying the insertion was amplified by PCR from M228 using primers PSA198/199. Following the SacB‐based mutagenesis method described above, the corresponding sequence carrying the inserted sequence was deleted from M227 resulting in a mutant designated M227Δ*DDE.* For the insertion of a *gfp* gene in M228 in the same site upstream of the *hrpR* gene in the T3SS cluster, a 2279 bp fusion DNA fragment containing a 759 bp upstream flank (using primers PSA259/260), an 841 bp *gfp* fragment amplified from pDSK‐GFPuv (using primers gfpF/R) (Wang *et al*., 2007), and a 678 bp downstream flank (using primers PSA263/264), was constructed by crossover‐PCR. Then, the fragment replaced the corresponding sequence in M228 as described above, resulting in a mutant designated M228*gfp*. The mutant was confirmed by PCR using PSA198/199 and fluorescence using an Olympus BX51 fluorescence microscope (Olympus, Melville, NY, USA).

Following the *SacB*‐based mutagenesis method described above, we individually performed the *in‐frame* deletion of the *tatC* gene, *copA* gene, *hopZ5* gene, *hrpH*‐*hrpZ5* cluster (designated as E cluster). With the primers listed in Table S2, the 5'‐ and 3'‐ end fragments of each gene/cluster were PCR amplified and fused as a single fragment. The fused fragments containing the upstream and downstream region of the targeting gene/cluster were digested with *EcoRI* and* BamHI* and cloned into the *EcoRI*/*HindIII* sites of pK18*mobSacB*. The recombinant plasmids were then transformed into *E. coli* strain S17‐1/λ_pir_ and sequenced. The kanamycin‐resistant Psa strains with each of the recombinant plasmids were obtained via conjugation and subsequently plated onto LB plates containing 15% sucrose to counter‐select the integration. The mutants M228Δ*tatC*, M228Δ*copA*, M228Δ*E* and M256Δ*hopZ5* were first screened by PCR with the primers listed in Table S2 and further confirmed by Sanger sequencing.

The four genes *tatC*,* copA*, *hopZ5* and *hopH1* containing the ribosome‐binding site from M228 were PCR cloned into pML123 under* nptII* promoter, a broad‐host‐range cloning vector with Gm resistance (Labes *et al*., 1990). The resulting plasmids p‐tatC and p‐copA were transformed by electroporation into M228Δ*tatC* and M228Δ*copA*, respectively. The resulting plasmids p‐hopZ5 and p‐hopH1 were individually transformed by electroporation into M228ΔE. Following the same procedure, five additional *copA* variants (*copA*_clade1_, *copA*_clade3_, *copA*_ICE‐NZ45_, *copA*_ICE‐C15_ and *copA*_pPsaNZ65_) were cloned into pML123 and transformed by electroporation into M228Δ*copA*.

### *Agrobacterium tumefaciens* infiltration and confocal microscopy analysis

*Agro*‐infiltration assays were carried out following the previously described procedure (Li *et al*., 2015). For transient expression, *A. tumefaciens* strain GV3101 carrying an expression plasmid (pGR106 : effector) was cultured and infiltrated into the upper leaves of 4‐week‐old *N. benthamian*a plants. Cell death symptoms were evaluated and photographed 3 days–4 days past infiltration. Each assay was performed in triplicate. For the subcellular localization, HopZ5 was cloned into pCAMBIA1302 with a C‐terminally tagged with green fluorescent protein (GFP), and the construct was transformed into *A. tumefaciens* strain AGL1 by electroporation. Two days post‐*Agro*‐infiltration in 4‐week‐old *N. benthamiana *leaves, the GFP signals were then observed using an Olympus BX‐53 microscope (Olympus Corporation, Tokyo, Japan) (excitation filter 485 nm, dichromic mirror 510 nm, and barrier filter 520 nm).

### PCR and Sanger sequencing

All primers used for PCR in this study were synthetized by Invitrogen (Shanghai, China) and are listed in Table S2. PCR amplifications were performed in a S1000 thermal cycler (Bio‐Rad) using the NEB Q5^®^ Hi‐Fi polymerase following the manufacturer’s instructions. Sanger sequencing was performed at Beijing AuGCT Biotechnology Co. Ltd. (China).

### Statistical analysis

Distributions of the three Psa3 clades might be associated with host varieties, host tissues, geographical origin and isolation time. Principal component analysis, stepwise discriminant analysis and chi‐square tests were performed for these factors using SPSS 19.0 software (IBM, Armonk, New York, USA). Analysis of variance (ANOVA) was performed for comparisons in pathogenicity between strains using SPSS 19.0.

## GenBank accession numbers

MDXF01, MDXG01, MDXH01, SNQS01, SNQT01, SNQU01, SNQV01.

## Supporting information


**Fig. S1** Maximum Likelihood tree of *Pseudomonas syringae* pv. *actinidiae* (Psa) biovars. The tree was constructed from a multi locus sequence analysis (MLSA) based on five concatenated house keeping genes (*gyrB* *rpoD* *pgi* *acnB* *cts*). Data were analysed by MEGA 6.06 with the Tamura Nei model. MP and NJ analyses produced a similar topology. Additional *Pseudomonas* strains were included for comparison. The bar indicates the sequence divergence. The solid triangle indicates Psa strains from Shaanxi province, China.

**Fig. S2** The reliability of the wound‐inoculation method was further evaluated by inoculating M228 on nine kiwifruit cultivars. The patterns in disease resistance of the nine cultivars were largely consistent with those observed in kiwifruit orchards (Qin *et al*., 2013). Cultivars of *Actinidiae chinensis* var. *chinensis*: ‘Hongyang’ (red flesh), ‘Xixuan’ (gold flesh) and ‘Jinyan’ (gold flesh); cultivars of *A. chinensis* var.* deliciosa*:  Hayward’, ‘Cuixiang’, ‘Qinmei’, ‘Yate’ and ‘Xuxiang’; hybrids between var. *chinensis* and var. *deliciosa*: ‘Huayou’ (gold flesh, main characteristics similar to var. *chinensis*).

**Fig. S3** The *copA* gene is required for copper resistance in* Pseudomonas syringae* pv. *actinidiae* (A, B, C); the  T259A  mutation of HopZ5 doesn t affect the subcellular localization and induction of HR like cell death (HCD) in *Nicotian* *benthamiana* leaves (D, E), and also showed no effect on host preference (F). (A) The minimal inhibitory concentration (MIC) of CuSO_4_ for clade 2 strain M228, M228Δ*copA*, and a series of constructs expressing *copA* gene in M228Δ*copA* determined by the agar‐dilution method. An *in‐frame* deletion of either the *copA* gene resulted in increased susceptibility to CuSO_4_, while the changed phenotype could be fully complemented by expressing *copA* gene cloned from other Psa3 clades, ICE‐ or plasmid‐borne *copABCD* operon. (B) The MIC of CuSO_4_ for three Psa3 clades isolated from diseased kiwifruit tissues. There are significant differences between clades (Duncan’s multiple range test, *P*<0.05). Experiments were repeated at least twice with similar results. (C) The Maximum‐Likelihood tree for the chromosomal *copA* gene in Psa and the ICE‐ or plasmid‐borne *copA *in* copABCD* operon. Three *copA* homologues marked by star symbol (plasmid‐borne *copA*_C15_ and *copA*_NZ65_, and ICE‐borne *copA*_NZ45_) were expressed in M228Δ*copA*. (D) HopZ5_clade3_ localized to the cell periphery in *N.* *benthamiana* leaves. Four week‐old *N. benthamiana* leaf cells were infected with *Agrobacterium* *tumefaciens* strain AGL1 carrying C‐terminally GFP‐tagged HopZ5_clade3_ effector for transient protein expression. (E) HopZ5_clade3_ triggered HCD in *N.* *benthamiana* leaves. Four week‐old *N. benthamiana* leaf cells were infected with *A. tumefaciens* strain GV3101 carrying *pGR106:Bax*. *pGR106:eGFP* and *pGR106:hopZ5*_clade3_. (F)The* hopZ5*_clade3_ deletion mutant showed significantly reduced pathogenicity on canes of *A. chinensis* var. *chinensis *‘Hongyang’, but not on canes of *A. chinensis* var. *deliciosa *‘Hayward’. The lesion length measured at 15 dpi of each mutant was normalized to that of M228. The experiments were repeated three times with similar results.

**Fig. S4** Seven conserved loci are present in the 1076‐bp noncoding sequence between *hrpH* and *hrpR* in the type III secretion system cluster from *Pseudomonas syringae*. An 1176 bp transposable element (*DDE1*) is inserted in CL2 in the low‐virulent *P. syringae* pv. *actinidiae* strain M227. The conserved motifs identified by alignment of 29 homologous sequences from diverse *P. syringae* members were shown using the MEME tool (Bailey *et al*., 2009). The asterisk indicates the location of the *DDE1* insertion, and the black arrows indicate the binding sites of the transcriptional activator cvsR of the *hrpR/S* operon. The illustration was drawn using the IBS tool (Liu *et al*., 2015).

**Fig. S5** Transcriptional patterns of the type III secretion system (T3SS) and effector genes in M228 and the mutant M228*gfp* in *hrp*‐inducing conditions. (1) M228*gfp* showed different transcriptional patterns of T3SS and effector genes from those of M228 in HDM. The nutrient rich KB medium and HDM medium are *hrp* repressing and *hrp* derepressing, respectively. Bacteria were cultured in KB at the designated  0 h , and subsequently transferred into HDM medium with a final concentration of 0.02 OD_600_ value. HrpR and HrpS transcriptionally regulate the *hrpL* gene, while HrpL serves as the global regulator of both the T3SS and T3E genes by targeting the upstream  *hrp* box  sequence. The genes *hrcC*, *hrpK1*, *hrczN*,* hrcQa *and *hrpZ* are in the T3SS cluster, and the 19 genes *hopM1, hopI1, avrE1, avrPto5, hopZ5, hopAZ1, hopAM1, hopF2, hopS2, hopAE1, hopZ3, hopY1, avrRpm1, hopN1, hopAU1, hopH1, hopR1, hopQ1 and hopD1* are effector genes. (2) M228*gfp* showed different transcriptional patterns of T3SS and effector genes from those of M228 *in planta*. M228 and M228*gfp* with 10^8^ cfu ml were inoculated on canes of *Actinidiae chinensis* var. *chinensis*: ‘Hongyang  using the wound inoculation method. Samples were collected at the designated time point. Quantitative real time PCR was performed in a BioRad IQ5 thermal cycler using SYBR Green reagent. Three replicates were performed for each sample. The results shown are the mean and standard deviation, and the relative expression ratios were compared among samples for each gene (Duncan s multiple range test, *P* 0.05). Experiments were repeated at least twice with similar results.

**Table S1** *Pseudomonas syringae* pv. *actinidiae* biovar 3 strains from Shaanxi Province, China used in this study.

**Table S2** Primers used in this study.

**Table S3** SNPs across the whole genomes of three Psa3 clade 2 strains.

**Table S4** Summary of the results of the comparative secretome analysis.
