# Supplementary figures and images for: Comparative genomics reveal pathogenicity‐related loci in Pseudomonas syringae pv. actinidiae biovar 3
Source: Mol Plant Pathol. 2019 Apr 26;20(7):923–42. doi: 10.1111/mpp.12803 (PMC6589868; doi:10.1111/mpp.12803)

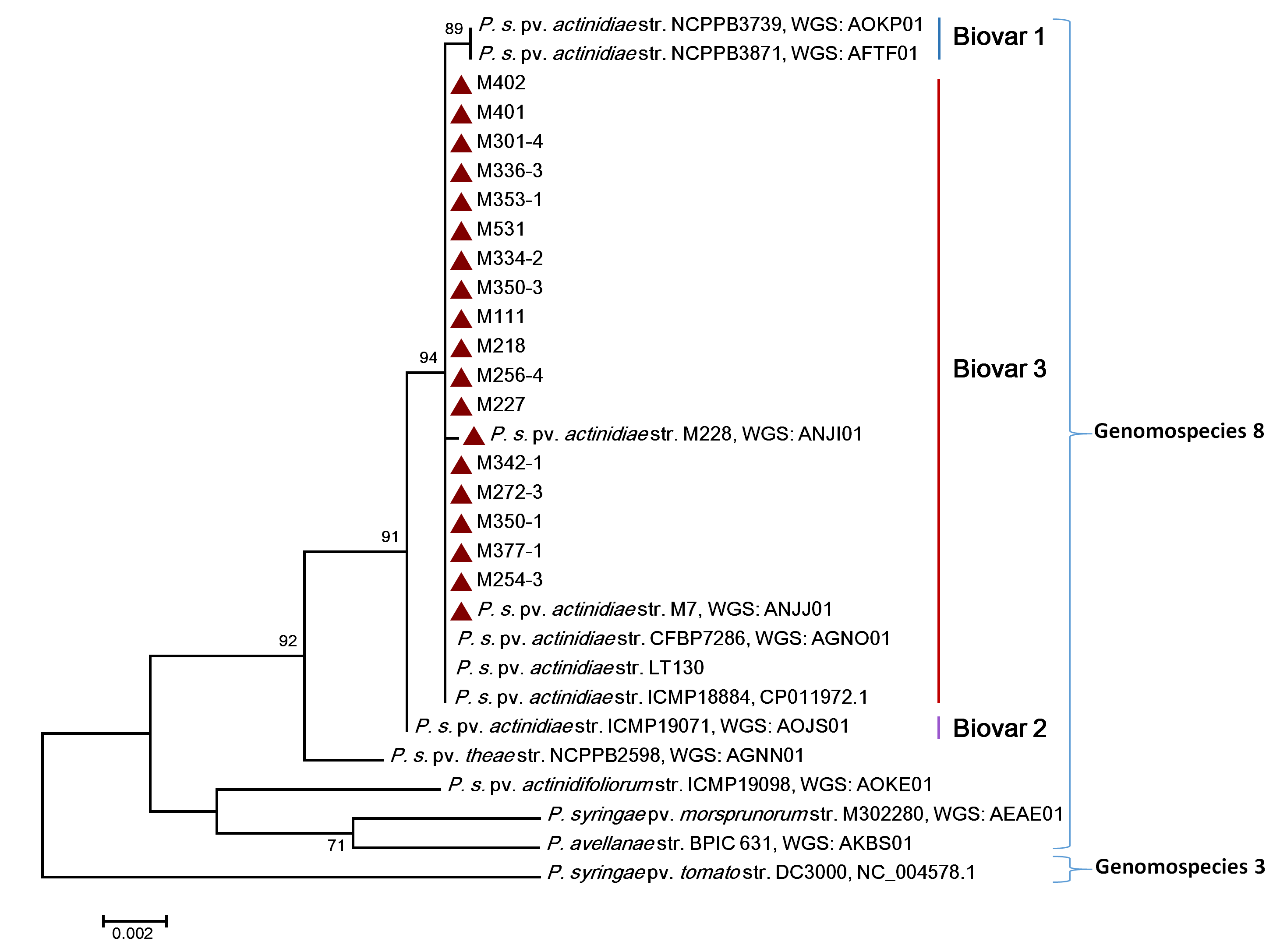

Supplement: Supplementary file 1 — Fig. S1 Maximum Likelihood tree of Pseudomonas syringae pv. actinidiae (Psa) biovars. The tree was constructed from a multi locus sequence analysis (MLSA) based on five concatenated house keeping genes (gyrB rpoD pgi acnB cts). Data were analysed by MEGA 6.06 with the Tamura Nei model. MP and NJ analyses produced a similar topology. Additional Pseudomonas strains were included for comparison. The bar indicates the sequence divergence. The solid triangle indicates Psa strains from Shaanxi province, China. [file MPP-20-923-s001.tif]

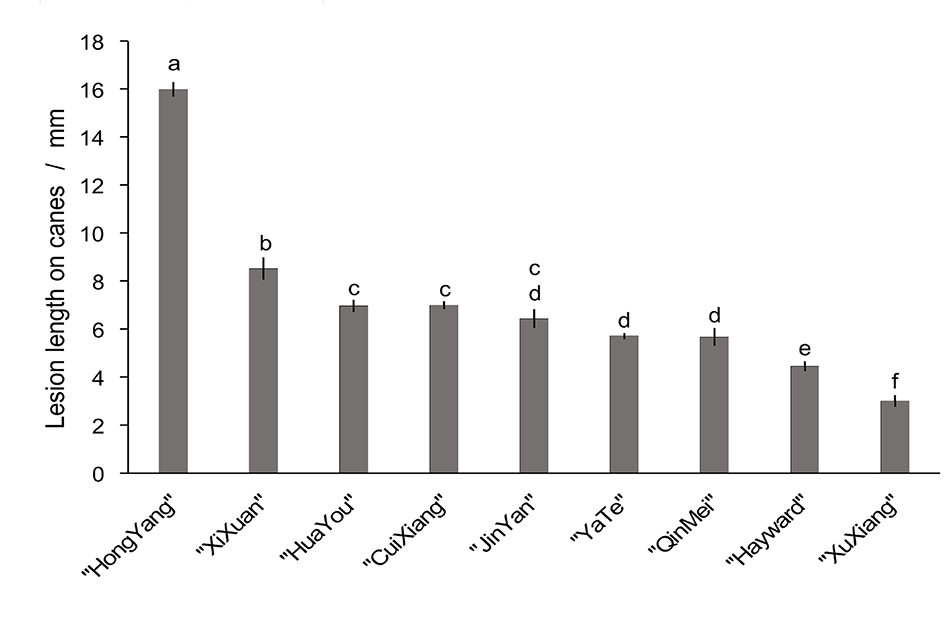

Supplement: Supplementary file 2 — Fig. S2 The reliability of the wound‐inoculation method was further evaluated by inoculating M228 on nine kiwifruit cultivars. The patterns in disease resistance of the nine cultivars were largely consistent with those observed in kiwifruit orchards (Qin et al., 2013). Cultivars of Actinidiae chinensis var. chinensis: ‘Hongyang’ (red flesh), ‘Xixuan’ (gold flesh) and ‘Jinyan’ (gold flesh); cultivars of A. chinensis var. deliciosa: Hayward’, ‘Cuixiang’, ‘Qinmei’, ‘Yate’ and ‘Xuxiang’; hybrids between var. chinensis and var. deliciosa: ‘Huayou’ (gold flesh, main characteristics similar to var. chinensis). [file MPP-20-923-s002.tif]

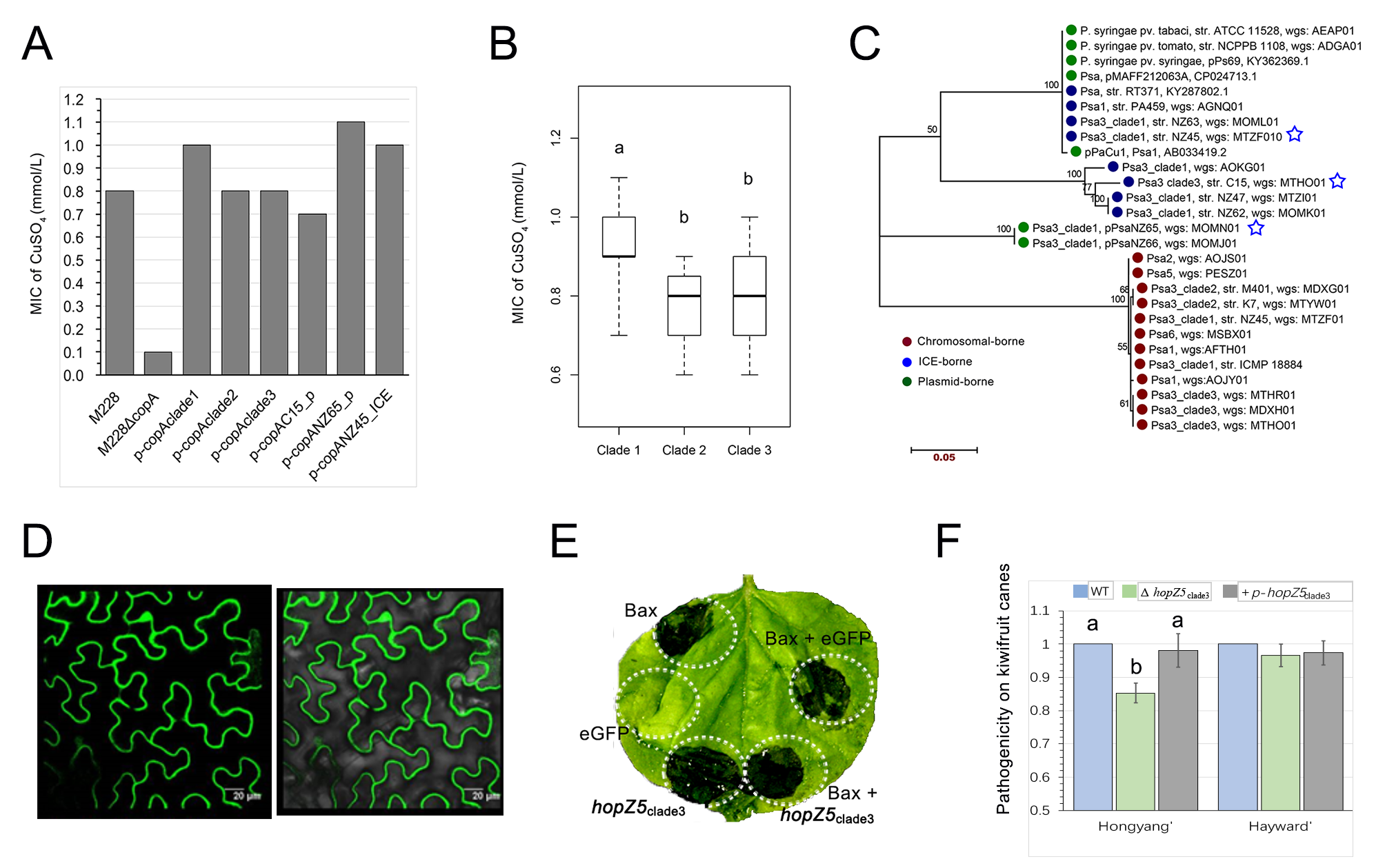

Supplement: Supplementary file 3 — Fig. S3 The copA gene is required for copper resistance in Pseudomonas syringae pv. actinidiae (A, B, C); the T259A mutation of HopZ5 doesn t affect the subcellular localization and induction of HR like cell death (HCD) in Nicotian benthamiana leaves (D, E), and also showed no effect on host preference (F). (A) The minimal inhibitory concentration (MIC) of CuSO4 for clade 2 strain M228, M228ΔcopA, and a series of constructs expressing copA gene in M228ΔcopA determined by the agar‐dilution method. An in‐frame deletion of either the copA gene resulted in increased susceptibility to CuSO4, while the changed phenotype could be fully complemented by expressing copA gene cloned from other Psa3 clades, ICE‐ or plasmid‐borne copABCD operon. (B) The MIC of CuSO4 for three Psa3 clades isolated from diseased kiwifruit tissues. There are significant differences between clades (Duncan’s multiple range test, P<0.05). Experiments were repeated at least twice with similar results. (C) The Maximum‐Likelihood tree for the chromosomal copA gene in Psa and the ICE‐ or plasmid‐borne copA in copABCD operon. Three copA homologues marked by star symbol (plasmid‐borne copA C15 and copA NZ65, and ICE‐borne copA NZ45) were expressed in M228ΔcopA. (D) HopZ5clade3 localized to the cell periphery in N. benthamiana leaves. Four week‐old N. benthamiana leaf cells were infected with Agrobacterium tumefaciens strain AGL1 carrying C‐terminally GFP‐tagged HopZ5clade3 effector for transient protein expression. (E) HopZ5clade3 triggered HCD in N. benthamiana leaves. Four week‐old N. benthamiana leaf cells were infected with A. tumefaciens strain GV3101 carrying pGR106:Bax. pGR106:eGFP and pGR106:hopZ5 clade3. (F)The hopZ5 clade3 deletion mutant showed significantly reduced pathogenicity on canes of A. chinensis var. chinensis ‘Hongyang’, but not on canes of A. chinensis var. deliciosa ‘Hayward’. The lesion length measured at 15 dpi of each mutant was normalized to that of M228. The experiments were repe [file MPP-20-923-s003.tif]

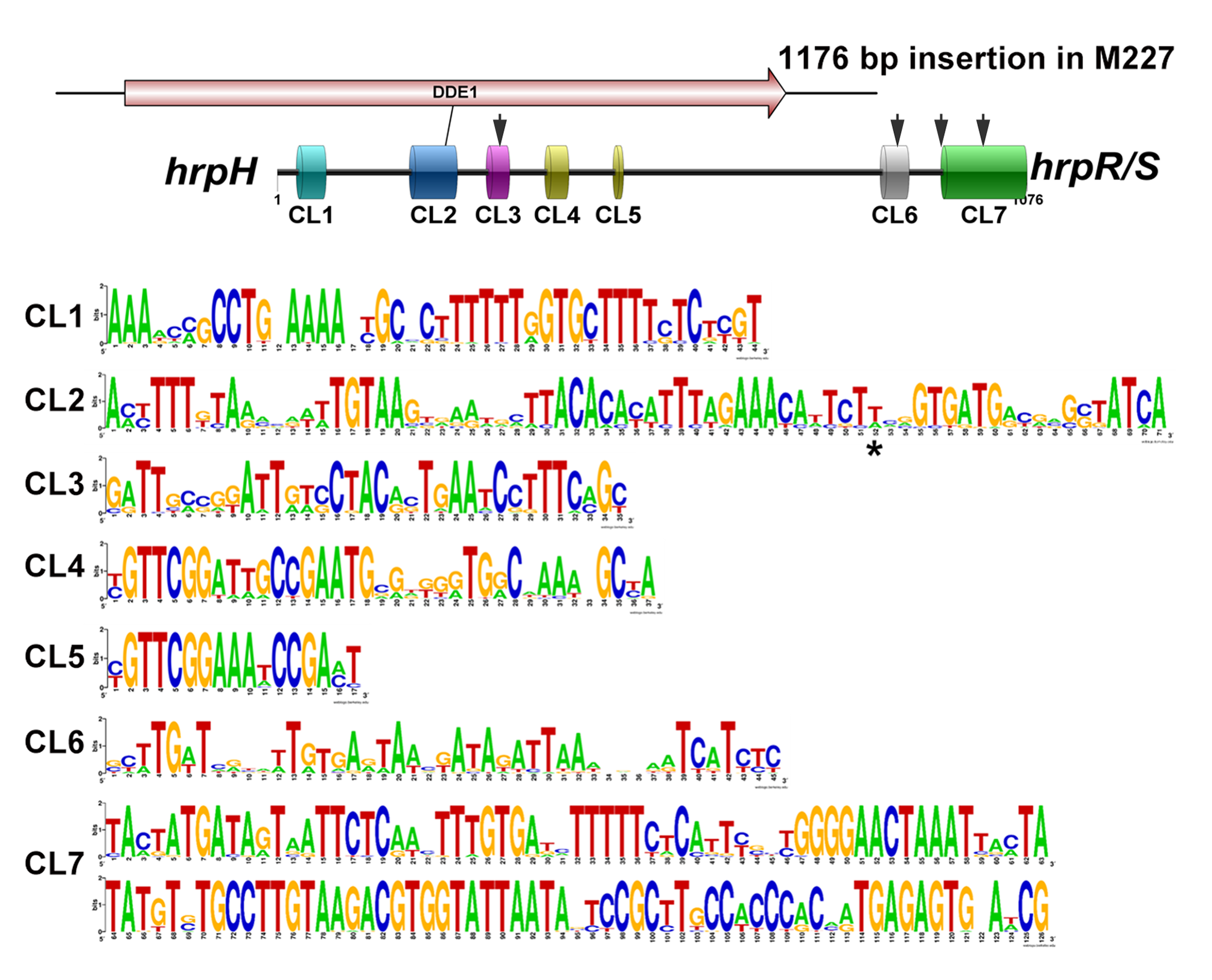

Supplement: Supplementary file 4 — Fig. S4 Seven conserved loci are present in the 1076‐bp noncoding sequence between hrpH and hrpR in the type III secretion system cluster from Pseudomonas syringae. An 1176 bp transposable element (DDE1) is inserted in CL2 in the low‐virulent P. syringae pv. actinidiae strain M227. The conserved motifs identified by alignment of 29 homologous sequences from diverse P. syringae members were shown using the MEME tool (Bailey et al., 2009). The asterisk indicates the location of the DDE1 insertion, and the black arrows indicate the binding sites of the transcriptional activator cvsR of the hrpR/S operon. The illustration was drawn using the IBS tool (Liu et al., 2015). [file MPP-20-923-s004.tif]

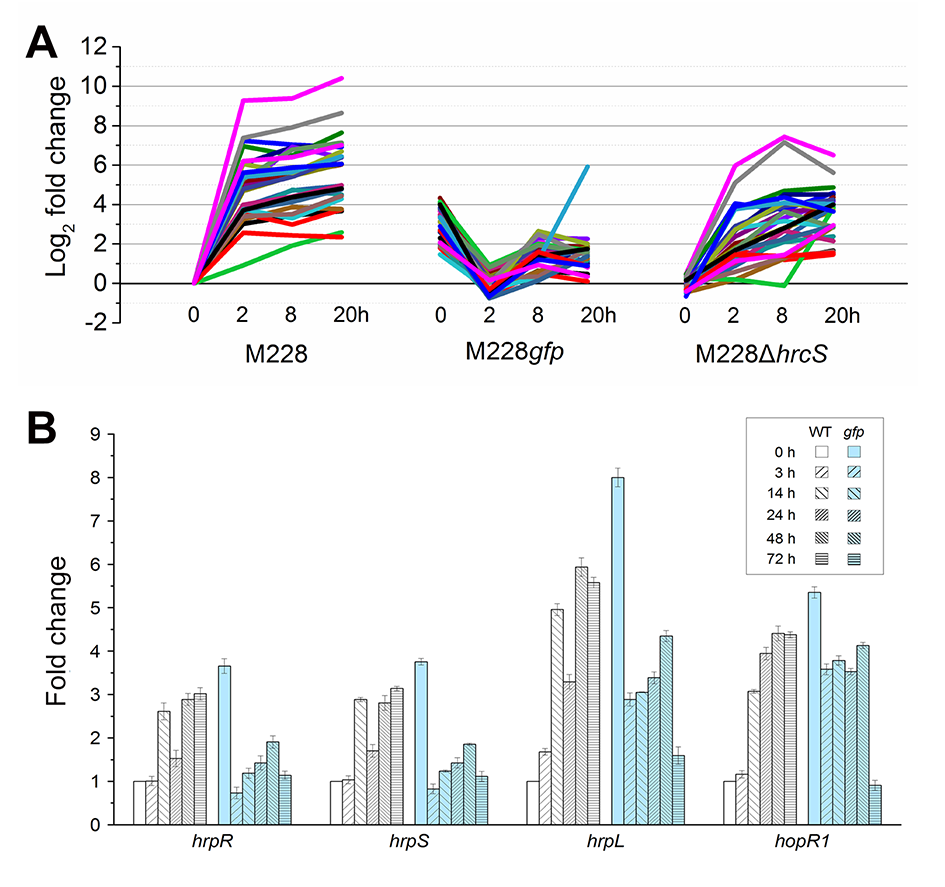

Supplement: Supplementary file 5 — Fig. S5 Transcriptional patterns of the type III secretion system (T3SS) and effector genes in M228 and the mutant M228gfp in hrp‐inducing conditions. (1) M228gfp showed different transcriptional patterns of T3SS and effector genes from those of M228 in HDM. The nutrient rich KB medium and HDM medium are hrp repressing and hrp derepressing, respectively. Bacteria were cultured in KB at the designated 0 h , and subsequently transferred into HDM medium with a final concentration of 0.02 OD600 value. HrpR and HrpS transcriptionally regulate the hrpL gene, while HrpL serves as the global regulator of both the T3SS and T3E genes by targeting the upstream hrp box sequence. The genes hrcC, hrpK1, hrczN, hrcQa and hrpZ are in the T3SS cluster, and the 19 genes hopM1, hopI1, avrE1, avrPto5, hopZ5, hopAZ1, hopAM1, hopF2, hopS2, hopAE1, hopZ3, hopY1, avrRpm1, hopN1, hopAU1, hopH1, hopR1, hopQ1 and hopD1 are effector genes. (2) M228gfp showed different transcriptional patterns of T3SS and effector genes from those of M228 in planta. M228 and M228gfp with 108 cfu ml were inoculated on canes of Actinidiae chinensis var. chinensis: ‘Hongyang using the wound inoculation method. Samples were collected at the designated time point. Quantitative real time PCR was performed in a BioRad IQ5 thermal cycler using SYBR Green reagent. Three replicates were performed for each sample. The results shown are the mean and standard deviation, and the relative expression ratios were compared among samples for each gene (Duncan s multiple range test, P 0.05). Experiments were repeated at least twice with similar results. [file MPP-20-923-s005.tif]
