# Supplementary material for: Comparative genomics reveal pathogenicity‐related loci in Pseudomonas syringae pv. actinidiae biovar 3
Source: Mol Plant Pathol. 2019 Apr 26;20(7):923–42. doi: 10.1111/mpp.12803 (PMC6589868; doi:10.1111/mpp.12803)
Supplement: Supplementary file 6 — Table S1 Pseudomonas syringae pv. actinidiae biovar 3 strains from Shaanxi Province, China used in this study. [file MPP-20-923-s006.docx]

**Table S1** *Pseudomonas syringae* pv. *actinidiae* biovar 3 strains from Shaanxi Province, China used in this study

| No. | Isolates^a^ | Host variety / cultivar^b^ | Host tissues | Isolation Date | Area^c^ | Clonal-complex^d^ |
| --- | --- | --- | --- | --- | --- | --- |
| 1 | M6 | *Actinidiae chinensis* var. *chinensis* ‘HongYang’ | Leaf | 2010/6/11 | 3 | 1 |
| 2 | M7 | *A. ch*. var. *chinense* ‘Hongyang’ | Leaf | 2010/6/11 | 3 | 1 |
| 3 | M23 | *A. ch*. var. *chinense* ‘Hongyang’ | Leaf | 2010/6/11 | 3 | 1 |
| 4 | M121 | *A. ch*. var. *chinense* ‘Hongyang’ | Cane | 2010/10/1 | 5 | 2 |
| 5 | JX1321 | *A. ch*. var. *deliciosa* ‘Hayward’ | Leaf | 2010/10/1 | 4 | 2 |
| 6 | M111 | *A. ch*. var. *deliciosa* ‘XuXiang’ | Leaf | 2010/10/19 | 1 | 3 |
| 7 | M208 | - | Cane | 2010/12/1 | 3 | 3 |
| 8 | M122 | *A. ch*. var. *chinense* ‘Hongyang’ | Cane | 2010/12/4 | 3 | 1 |
| 9 | M228 | *A. ch*. var. *chinense* ‘Hongyang’ | Cane | 2010/12/4 | 3 | 2 |
| 10 | M227 | *A. ch*. var. *deliciosa* ‘QinMei’ | Cane | 2010/12/4 | 3 | 2 |
| 11 | M218 | *A. ch*. var. *chinense* ‘XiXuan’ | Cane | 2010/12/5 | 2 | 2 |
| 12 | M240-1 | *A. ch*. var. *chinense* ‘Hongyang’ | Cane | 2011/2/21 | 3 | 1 |
| 13 | M241-1-1 | *A. ch*. var. *chinense* ‘Hongyang’ | Cane | 2011/2/21 | 3 | 1 |
| 14 | M247-3 | *A. ch*. var. *chinense* ‘Hongyang’ | Cane | 2011/3/8 | 3 | 3 |
| 15 | M251-2 | *A. ch*. var. *chinense* ‘Hongyang’ | Cane | 2011/3/8 | 1 | 3 |
| 16 | M252-1 | *A. ch*. var. *chinense* ‘Hongyang’ | Cane | 2011/3/8 | 1 | 3 |
| 17 | M258-2 | *A. ch*. var. *chinense* ‘Hongyang’ | Cane | 2011/3/8 | 3 | 2 |
| 18 | M265-4 | *A. ch*. var. *chinense* ‘Hongyang’ | Cane | 2011/3/8 | 3 | 2 |
| 19 | M265-5 | *A. ch*. var. *chinense* ‘Hongyang’ | Cane | 2011/3/8 | 3 | 1 |
| 20 | M271-4 | *A. ch*. var. *chinense* ‘Hongyang’ | Cane | 2011/3/8 | 3 | 1 |
| 21 | M272-3 | *A. ch*. var. *chinense* ‘Hongyang’ | Cane | 2011/3/8 | 1 | 1 |
| 22 | M277-5 | *A. ch*. var. *chinense* ‘Hongyang’ | Cane | 2011/3/8 | 3 | 2 |
| 23 | M254-3 | *A. ch*. var. *deliciosa* ‘Hayward’ | Cane | 2011/3/8 | 1 | 3 |
| 24 | M255-3 | *A. ch*. var. *deliciosa* ‘Hayward’ | Cane | 2011/3/8 | 1 | 2 |
| 25 | M256 | *A. ch*. var. *deliciosa* ‘Hayward’ | Cane | 2011/3/8 | 1 | 3 |
| 26 | M275-3 | *A. ch*. var. *deliciosa* ‘Hayward’ | Cane | 2011/3/8 | 1 | 3 |
| 27 | M266-4 | *A. ch*. var. *deliciosa* ‘QinMei’ | Cane | 2011/3/8 | 3 | 3 |
| 28 | M268-4 | *A. ch*. var. *deliciosa* ‘QinMei’ | Cane | 2011/3/8 | 3 | 3 |
| 29 | M269-2-1 | *A. ch*. var. *deliciosa* ‘QinMei’ | Cane | 2011/3/8 | 3 | 1 |
| 30 | M284-4 | - | Cane | 2011/3/13 | 1 | 1 |
| 31 | M292-2 | *A. ch*. var. *deliciosa* ‘Hayward’ | Cane | 2011/3/15 | 2 | 2 |
| 32 | M301-3 | *A. ch*. var. *deliciosa* ‘Hayward’ | Cane | 2011/3/15 | 2 | 3 |
| 33 | M301-4 | *A. ch*. var. *deliciosa* ‘Hayward’ | Cane | 2011/3/15 | 2 | 3 |
| 34 | M303-3 | ‘HuaYou’ | Cane | 2011/3/15 | 2 | 2 |
| 35 | M333-1 | *A. ch*. var. *chinense* ‘Hort16A’ | Leaf | 2011/6/8 | 1 | 2 |
| 36 | M333-3 | *A. ch*. var. *chinense* ‘Hort16A’ | Leaf | 2011/6/8 | 1 | 2 |
| 37 | M338-2 | *A. ch*. var. *deliciosa* ‘Hayward’ | Leaf | 2011/6/8 | 1 | 3 |
| 38 | M338-4 | *A. ch*. var. *deliciosa* ‘Hayward’ | Leaf | 2011/6/8 | 1 | 3 |
| 39 | M336-1 | *A. ch*. var. *deliciosa* ‘Xuxiang’ | Leaf | 2011/6/8 | 1 | 3 |
| 40 | M336-2 | *A. ch*. var. *deliciosa* ‘Xuxiang’ | Leaf | 2011/6/8 | 1 | 3 |
| 41 | M336-3 | *A. ch*. var. *deliciosa* ‘Xuxiang’ | Leaf | 2011/6/8 | 1 | 3 |
| 42 | M336-4 | *A. ch*. var. *deliciosa* ‘Xuxiang’ | Leaf | 2011/6/8 | 1 | 3 |
| 43 | M337-1 | *A. ch*. var. *deliciosa* ‘YaTe’ | Leaf | 2011/6/8 | 1 | 3 |
| 44 | M334-1 | Stock | Leaf | 2011/6/8 | 3 | 2 |
| 45 | M334-2 | Stock | Leaf | 2011/6/8 | 3 | 2 |
| 46 | M334-4 | Stock | Leaf | 2011/6/8 | 3 | 3 |
| 47 | M335-4 | Stock | Leaf | 2011/6/8 | 3 | 1 |
| 48 | M339-1 | Stock | Leaf | 2011/6/8 | 1 | 1 |
| 49 | M353-1 | *A. ch*. var. *chinense* ‘Hongyang’ | Leaf | 2011/10/21 | 3 | 1 |
| 50 | M353-2 | *A. ch*. var. *chinense* ‘Hongyang’ | Leaf | 2011/10/21 | 3 | 1 |
| 51 | M348-2 | *A. ch*. var. *chinense* ‘Hongyang’ | Cane | 2011/10/21 | 3 | 1 |
| 52 | M350-1 | *A. ch*. var. *chinense* ‘Hongyang’ | Cane | 2011/10/21 | 3 | 1 |
| 53 | M350-3 | *A. ch*. var. *chinense* ‘Hongyang’ | Cane | 2011/10/21 | 3 | 1 |
| 54 | M346-1 | *A. ch*. var. *deliciosa* ‘Xuxiang’ | Cane | 2011/10/21 | 3 | 1 |
| 55 | M342-1 | *A. ch*. var. *chinense* ‘Xixuan’ | Cane | 2011/10/27 | 2 | 2 |
| 56 | M342-2 | *A. ch*. var. *chinense* ‘Xixuan’ | Cane | 2011/10/27 | 2 | 2 |
| 57 | M342-3 | *A. ch*. var. *chinense* ‘Xixuan’ | Cane | 2011/10/27 | 2 | 2 |
| 58 | M343-1 | *A. ch*. var. *chinense* ‘Xixuan’ | Cane | 2011/10/27 | 2 | 2 |
| 59 | M343-3 | *A. ch*. var. *chinense* ‘Xixuan’ | Cane | 2011/10/27 | 2 | 2 |
| 60 | M344-2 | *A. ch*. var. *chinense* ‘Xixuan’ | Cane | 2011/10/27 | 2 | 2 |
| 61 | M378-4 | *A. ch*. var. *deliciosa* ‘Hayward’ | Cane | 2012/3/12 | 1 | 3 |
| 62 | M371 | *A. ch*. var. *chinense* ‘Hongyang’ | Cane | 2012/3/13 | 1 | 3 |
| 63 | M372-1 | *A. ch*. var. *chinense* ‘Hongyang’ | Cane | 2012/3/13 | 3 | 1 |
| 64 | M373-2 | *A. ch*. var. *chinense* ‘Hongyang’ | Cane | 2012/3/13 | 3 | 3 |
| 65 | M374-1 | *A. ch*. var. *chinense* ‘Hongyang’ | Cane | 2012/3/13 | 3 | 1 |
| 66 | M374-2 | *A. ch*. var. *chinense* ‘Hongyang’ | Cane | 2012/3/13 | 3 | 1 |
| 67 | M369 | *A. ch*. var. *chinense* ‘Hort16A’ | Cane | 2012/3/13 | 1 | 1 |
| 68 | M376-1 | *A. ch*. var. *deliciosa* ‘Hayward’ | Cane | 2012/3/13 | 1 | 2 |
| 69 | M377-1 | *A. ch*. var. *deliciosa* ‘Hayward’ | Cane | 2012/3/13 | 1 | 3 |
| 70 | M378-1 | *A. ch*. var. *deliciosa* ‘Hayward’ | Cane | 2012/3/13 | 1 | 3 |
| 71 | M380-2 | *A. ch*. var. *deliciosa* ‘Hayward’ | Cane | 2012/3/13 | 1 | 3 |
| 72 | M367-1 | *A. ch*. var. *deliciosa* ‘Qinmei’ | Cane | 2012/3/13 | 1 | 2 |
| 73 | M368-1 | *A. ch*. var. *deliciosa* ‘Qinmei’ | Cane | 2012/3/13 | 1 | 2 |
| 74 | M368-2 | *A. ch*. var. *deliciosa* ‘Qinmei’ | Cane | 2012/3/13 | 1 | 2 |
| 75 | M375-1 | *A. ch*. var. *deliciosa* ‘Xuxiang’ | Cane | 2012/3/13 | 3 | 1 |
| 76 | M375-2 | *A. ch*. var. *deliciosa* ‘Xuxiang’ | Cane | 2012/3/13 | 3 | 1 |
| 77 | M364-1 | ‘Huanyou’ | Cane | 2012/3/13 | 1 | 2 |
| 78 | M365-1 | ‘Huanyou’ | Cane | 2012/3/13 | 1 | 3 |
| 79 | M366 | ‘Huanyou’ | Cane | 2012/3/13 | 1 | 3 |
| 80 | M383 | *A. ch*. var. *chinense* ‘Hongyang’ | Leaf | 2012/5/4 | 3 | 1 |
| 81 | M384 | *A. ch*. var. *deliciosa* ‘Hayward’ | Leaf | 2012/5/4 | 1 | 3 |
| 82 | M386 | *A. ch*. var. *deliciosa* ‘Hayward’ | Leaf | 2012/5/4 | 1 | 3 |
| 83 | M401 | *A. ch*. var. *chinense* ‘Hort16A’ | Cane | 2012/5/15 | 2 | 2 |
| 84 | M402 | *A. ch*. var. *chinense* ‘Hort16A’ | Cane | 2012/5/15 | 2 | 2 |
| 85 | M403 | *A. ch*. var. *chinense* ‘Hort16A’ | Cane | 2012/5/15 | 2 | 1 |
| 86 | M500-2 | *A. ch*. var. *chinense* ‘Hongyang’ | Cane | 2013/1/15 | 2 | 1 |
| 87 | M522 | *A. ch*. var. *chinense* ‘Hongyang’ | Cane | 2013/3/5 | 2 | 2 |
| 88 | M526 | *A. ch*. var. *chinense* ‘Hort16A’ | Root | 2013/3/5 | 2 | 2 |
| 89 | M529 | *A. ch*. var. *chinense* ‘Hongyang’ | Cane | 2013/3/15 | 3 | 2 |
| 90 | M530 | *A. ch*. var. *chinense* ‘Hongyang’ | Cane | 2013/3/15 | 3 | 2 |
| 91 | M531 | *A. ch*. var. *chinense* ‘Hongyang’ | Cane | 2013/3/15 | 3 | 2 |
| 92 | M532 | *A. ch*. var. *chinense* ‘Hongyang’ | Cane | 2013/3/15 | 3 | 2 |
| 93 | M591 | *A. ch*. var. *deliciosa* ‘Qinmei’ | Cane | 2013/3/24 | 4 | 1 |
| 94 | M611 | *A. ch*. var. *deliciosa* ‘Qinmei’ | Cane | 2013/3/24 | 5 | 1 |
| 95 | M615 | *A. ch*. var. *deliciosa* ‘Qinmei’ | Cane | 2013/3/24 | 5 | 1 |
| 96 | M588 | *A. ch*. var. *deliciosa* ‘Xuxiang’ | Cane | 2013/3/24 | 4 | 3 |
| 97 | M608 | ‘Huanyou’ | Cane | 2013/3/24 | 5 | 1 |
| 98 | M407-1 | *A. ch*. var. *chinense* ‘Hongyang’ | Cane | 2014/7/23 | 3 | 2 |
| 99 | M407-2 | *A. ch*. var. *chinense* ‘Hongyang’ | Cane | 2014/7/23 | 3 | 2 |
| 100 | M407-3 | *A. ch*. var. *chinense* ‘Hongyang’ | Cane | 2014/7/23 | 3 | 2 |
| 101 | M407-4 | *A. ch*. var. *chinense* ‘Hongyang’ | Cane | 2014/7/23 | 3 | 2 |
| 102 | M407-5 | *A. ch*. var. *chinense* ‘Hongyang’ | Cane | 2014/7/23 | 3 | 2 |
| 103 | M20150301 | - | Cane | 2015/3/20 | 2 | 2 |
| 104 | M20150403 | - | Flower | 2015/4/29 |  | 1 |
| 105 | M20150401 | - | Leaf | 2015/4/29 |  | 1 |
| 106 | M20150402 | - | Leaf | 2015/4/29 |  | 1 |

1. This list include Shaanxi Psa3 strains listed in Table 1.
2. Cultivars of *Actinidiae chinensis* var. *chinensis*: ‘HongYang’, ‘Hort16A’ and ‘Xixuan’; cultivars of *A. chinensis* var. *deliciosa*: ‘Hayward’, ‘Xuxiang’, ‘Qinmei’ and ‘Yate’; ‘Huayou’ is the hybrids between var. *chinensis* and var. *deliciosa* showing characters similar to var. *chinensis*.

**c.** Area, in a radius of about 10 kilometers.

**d.** Clonal-complex is designated in this study.
