# Supplementary material for: Comparative genomics reveal pathogenicity‐related loci in Pseudomonas syringae pv. actinidiae biovar 3
Source: Mol Plant Pathol. 2019 Apr 26;20(7):923–42. doi: 10.1111/mpp.12803 (PMC6589868; doi:10.1111/mpp.12803)
Supplement: Supplementary file 7 — Table S2 Primers used in this study. [file MPP-20-923-s007.docx]

**Table S2** Primers used in this study

| No. | Primer pairs | Primer sequence (5' - 3') | Product size (bp)^a^ | | | Gene/protein^c^ | Used for: | Reference |
| --- | --- | --- | --- | --- | --- | --- | --- | --- |
| 1 | Tae4-Fd | ggaattccatatgacacggccattttttactg | 1673 | 607 | 501 | Tae4, putative T6SS substrate | Psa3 clustering^b^ | This study |
|  | Tae4-Rd | gcgctcgagtgtgaggctccagaaattcg |  |  |  |  |  |  |
| 2 | Tai4-2-Fd | atgaggctatctcttgcaatc | 2106 | 436 | 435 | Tai4-2, putative T6SS substrate | Psa3 clustering | This study |
|  | Tai4-2-Rd | tcatttatagtcttgcatataggtat |  |  |  |  |  |  |
| 3 | hopAW1-F | ttcccgggtatgcgcgtgagagtatcaaac | 663 | 671 | 2275 | HopAW1, T3E | Psa3 clustering | This study |
|  | hopAW1-R | tcgcgtcgacttacgagcgcacaggcag |  |  |  |  |  |  |
| 4 | hopAG1-Fd | gagcgtattaaggcactctgtg | 590 | 696 | 590 | HopAG1, T3E | Psa3 clustering | This study |
|  | hopAG1-Rd | cgtgatgtcgttcggtcgt |  |  |  |  |  |  |
| 5 | hopAC1-Fd | aatgtctaccagggcgtagtgc | 2004 | 330 | 330 | HopAC1, discontinued T3E | Psa3 clustering | This study |
|  | hopAC1-Rd | cgtgcggtgaatccaagtgt |  |  |  |  |  |  |
| 6 | hopR1-Fd | cccgagacgagactgttcacc | 0 | 1441 | 0 | HopR1, T3E | Psa3 clustering | This study |
|  | hopR1-Rd | tcgcccatatcgcccat |  |  |  |  |  |  |
| 7 | 12700-Fd | gcggttgctggcggtttgaa | 2259 | 429 | 2259 | Retron-type reverse transcriptase | Psa3 clustering | This study |
|  | 12700-Rd | gcggttctgcgatgtcagttgg |  |  |  |  |  |  |
| 8 | PsaF | cagaggcgctaacgaggaaa | 311 | | | *HopZ3* | Psa-specific | Balestra et al., 2013 |
|  | PsaR | cgagcatacatcaacaggtca |  |  |  |  |  |  |
| 9 | KNF | cacgatacatgggcttatgc | 492 | | | Putative lipoprotein | Psa-specific | Koh and Nou, 2002 |
|  | KNR | cttttcatccacacactccg |  |  |  |  |  |  |
| 10 | PsaF1 | ttttgctttgcacacccgatttt | 280 | | | ITS | Psa-specific | Rees-George et al., 2010 |
|  | PsaR2 | cacgcacccttcaatcaggatg |  |  |  |  |  |  |
| 11 | acnF | acatcccgytgcacgchctgkcc | 673 | | | *AcnB* | MLSA^c^ | Zhao et al. 2015 |
|  | acnR | gtggtgtcctgggavccbacggtg |  |  |  |  |  |  |
| 12 | ctsF | agttgatcatcgagggcgchgcc | 618 | | | *GltA* | MLSA | Zhao et al. 2015 |
|  | ctsR | tgatcggtttgatctcgcacgg |  |  |  |  |  |  |
| 13 | gyrB-F | gggcggyaagttcgaygacaaytc | 665 | | | *GyrB* | MLSA | Zhao et al. 2015 |
|  | gyrB-R | taatbgcrgtcarrccttcrcgsgc |  |  |  |  |  |  |
| 14 | pgi-Fp | tgcagrayttcagcatgcgcgadgc | 632 | | | *Pgi* | MLSA | Zhao et al. 2015 |
|  | pgi-Rp | cgagccgccctgbgccagrtaccag |  |  |  |  |  |  |
| 15 | rpoD-Fp | aaggcgaratcgaaatcgccaarcg | 582 | | | *RpoD* | MLSA | Zhao et al. 2015 |
|  | rpoD-Rp | ggaactgscgcaggaagtcggcrcg |  |  |  |  |  |  |
| 16 | hfq-F | cgcggatcccacggattcaccccaacg | 1860 | | | *Hfq* | *Hfq* swap | This study |
|  | hfq-R | gcgaagcttctccacgccttcaagcaga |  |  |  |  |  |  |
| 17 | hfq-DF | caaatcttgctatcgctccg | 663 | | | *Hfq* | *Hfq* swap, mutant detection | This study |
|  | hfq-DR | aggcgaatcgggcgaac |  |  |  |  |  |  |
| 18 | tatC-NO | gcggaattccgaggactgtttcatgggta | 790 | | | upstream region | In-frame deletion of *tatC* | This study |
|  | tatC-NI | ccatccactaaacttaaacacggcatttgctgatcgtt |  |  |  |  |  |  |
| 19 | tatC-CI | tgtttaagtttagtggatggggacgctgacaagcagga | 866 | | | downstream region |  | This study |
|  | tatC-CO | cgcggatccgcgagatgttgagataacccac |  |  |  |  |  |  |
| 20 | tatC-DO-F | aactacgcctggactgtgatg | WT, 3381 bp; Mutant, 2661 bp | | | *tatC* | Mutant detection | This study |
|  | tatC-DO-R | ggatgttgtctgccttgagc |  |  |  |  |  |  |
| 21 | tatC-F | gcgaagcttcgtcattgccaccgc | 840 | | | *tatC* | Expression in pML123 | This study |
|  | tatC-R | gcgctcgagcaagtaacagcaggttcacgg |  |  |  |  |  |  |
| 22 | copA-NO | gcggaattcgggaccttcatgtgca | 1078 | | | upstream region | In-frame deletion of *copA* | This study |
|  | copA-NI | aggtgcgtcttgatgttcg |  |  |  |  |  |  |
| 23 | copA-CI | cgcgggtggaagaatgagg | 680 | | | downstream region |  | This study |
|  | copA-CO | cgccagtgggttgaagg |  |  |  |  |  |  |
| 24 | copA-F | gcgaagcttctgaccggatacctcacca | WT, 1803 bp; Mutant, 42 bp | | | *copA* | Mutant detection & gene expression | This study |
|  | copA-R | gcgctcgagaagtcctcattcttccaccc |  |  |  |  |  |  |
| 25 | E-NO | ggtggatccccgtctgcatttatgtggaa | 505 | | | upstream region | In-frame deletion of *hopZ5-hopH1* cluster | This study |
|  | E-NI | cctgagcggcgtaagtattgtagcctgaagttgt |  |  |  |  |  |  |
| 26 | E-CI | caatacttacgccgctcagggctaatcat | 1159 | | | downstream region |  | This study |
|  | E-CO | ttatccgcttcggtggg |  |  |  |  |  |  |
| 27 | HopH1-F | gcgaagcttgaacaccaataggctaaatctcaa | WT, 866 bp; Mutant, 0 | | | *hopH1* ORF | Mutant detection & gene expression | This study |
|  | HopH1-R | gcgctcgaggaacgcccagtcaaatcg |  |  |  |  |  |  |
| 28 | HopZ5-F | gcgaagcttacatcatggacggaaccttt | WT, 1114 bp; Mutant, 0 | | | *hopZ5* ORF | Mutant detection & gene expression | This study |
|  | HopZ5-R | gcgctcgaggctttaggattctatcgcttttc |  |  |  |  |  |  |
| 29 | SacB-F | gcaaacactggaactgaagatgg | 478 | | | *SacB* gene in pK18*mobSacB* |  | This study |
|  | SacB-R | ttcctttcgcttgaggtacagc |  |  |  |  |  |  |
| 30 | PSA198 | tacgaattcaaaagaccttcgatggacc | 1470 | | | T3SS cluster | M227-specfic variant detection, locus swap | This study |
|  | PSA199 | gccaagcttgaccccgccgatagact |  |  |  |  |  |  |
| 31 | PSA184 | caccgtccgggtacaac | 1292 | | | Hypothetical protein | Ditto | This study |
|  | PSA185 | gaaaactacgcacagtccaaa |  |  |  |  |  |  |
| 32 | PSA188 | aaaagaccttcgatggacc | 1380 | | | DNA ligase-associated DEXH box helicase | Ditto | This study |
|  | PSA189 | gaccccgccgatagact |  |  |  |  |  |  |
| 33 | PSA194 | cgatggagtgaatgggc | 1398 | | | DNA-binding response regulator | Ditto | This study |
|  | PSA195 | cggtgaagggcgagata |  |  |  |  |  |  |
| 34 | hfq-orf-F | cttaaaggagtgcggcacatg | 279 | | | RNA-binding protein Hfq | Ditto | This study |
|  | hfq-orf-R | tcaggcgttacctggctcg |  |  |  |  |  |  |
| 35 | PSA176 | tcattaccccgaacgacg | 1476 | | | DNA-directed RNA polymerase subunit beta | Ditto | This study |
|  | PSA177 | ggctttcttgccgcttt |  |  |  |  |  |  |
| 36 | PSA200 | aaccgtctatgtcaccaactt | 1496 | | | Non-coding sequence | Ditto | This study |
|  | PSA201 | cggccaaacgaaatcct |  |  |  |  |  |  |
| 37 | PSA202 | aacccgacaaatcaccct | 1428 | | | Lytic transglycosylase | Ditto | This study |
|  | PSA203 | caccggcacttctttactg |  |  |  |  |  |  |
| 38 | PSA259 | tacgaattcgtctgctgatacccaaaa | 759 | | | Upstream flank of the ‘-930’ locus | *gfp* insertion at -930 bp upstream of *hrpR* | This study |
|  | PSA260 | attgcagttcacctagaatgtttctaaatgtgt |  |  |  |  |  |  |
| 39 | gfp-F | cattctaggtgaactgcaattttagagagacgg | 841 | | | A DNA fragment containg GFPuv gene | *gfp* insertion at -930 bp upstream of *hrpR* | This study |
|  | gfp-R | atcacctagcaccatgattacgccaagct |  |  |  |  |  |  |
| 40 | PSA263 | taatcatggtgctaggtgatggcacgctatc | 678 | | | Downstream flank of the ‘-930’ locus | *gfp* insertion at -930 bp upstream of *hrpR* | This study |
|  | PSA264 | gccaagcttgcaggctcttgagatgattacttt |  |  |  |  |  |  |
| 41 | 6600NO | cgcggatcctggagtttgaacacgaggacg | 1527 | | | *hrcC* | In-frame deletion of *hrcC* | This study |
|  | 6600NI | gccatccactaaacttaaacacatttcacactcccggttgc |  |  |  |  |  |  |
| 42 | 6600CI | tgtttaagtttagtggatggcggagcgaaaccatgatgattc | 1532 | | | *hrcC* | In-frame deletion of *hrcC* | This study |
|  | 6600CO | cccaagcttgccgtcggtcacatctacaa |  |  |  |  |  |  |
| 43 | 6600-DF | agtctcggtgacctgctcg | WT-2592;  Mutant-531 | | | *hrcC* | Detection of *hrcC* mutant | This study |
|  | 6600-DR | cgcggccttctatgtgc |  |  |  |  |  |  |
| 44 | gyrA-RT-F | aacattccgccgcataacc | 234 | | | *gyrA* | Psa qRT-PCR, reference gene | This study |
|  | gyrA-RTR | ctgacgaccgcccacctt |  |  |  |  |  |  |
| 45 | gyrB-RT-F | acccgaacgaagccaaagc | 201 | | | *gyrB* | Psa qRT-PCR, reference gene | This study |
|  | gyrB-RTR | atccgccagcagagtccc |  |  |  |  |  |  |
| 46 | PSA287 | cgatacggcttcatcatcattacc | 178 | | | *hopY1* | qRT-PCR | This study |
|  | PSA288 | tgtcggctttgtttgcttgg |  |  |  |  |  |  |
| 47 | PSA289 | cgccacatggagtcggtattg | 186 | | | *hopQ1* | qRT-PCR | This study |
|  | PSA290 | tgtcggtcgcattgttgtaagc |  |  |  |  |  |  |
| 48 | PSA291 | acccaccgtcacattagcga | 166 | | | *hopX3* | qRT-PCR | This study |
|  | PSA292 | gccagtagccattatcagacagttc |  |  |  |  |  |  |
| 49 | PSA293 | ggaactcaagcctatgcgttgt | 173 | | | *hopS2* | qRT-PCR | This study |
|  | PSA294 | ctggcggcgacagaataact |  |  |  |  |  |  |
| 50 | PSA295 | tcacccacattgcgtcttcatt | 230 | | | *hopI1* | qRT-PCR | This study |
|  | PSA296 | gtgcgtctcgacattgttcacc |  |  |  |  |  |  |
| 51 | PSA297 | gtgacggcaccacgcaata | 194 | | | *hopN1* | qRT-PCR | This study |
|  | PSA298 | gccataatcagcagcaaacg |  |  |  |  |  |  |
| 52 | PSA299 | cgggctcggtgtcttatctg | 271 | | | *hopM1* | qRT-PCR | This study |
|  | PSA300 | acggtatccacggccattt |  |  |  |  |  |  |
| 53 | PSA301 | cgcccaaagacaacgacaa | 289 | | | *avrE1* | qRT-PCR | This study |
|  | PSA302 | gccaacgtcccgatactgc |  |  |  |  |  |  |
| 54 | PSA303 | agccaaggactttgccattt | 214 | | | *avrRpm1* | qRT-PCR | This study |
|  | PSA304 | tgccgcacgcatttcat |  |  |  |  |  |  |
| 55 | PSA307 | cccgagacgagactgttcacc | 268 | | | *hopR1* | qRT-PCR | This study |
|  | PSA308 | gactgctttagccgttcccac |  |  |  |  |  |  |
| 56 | PSA309 | gcagacctgatggtggat | 217 | | | *hopAM1* | qRT-PCR | This study |
|  | PSA310 | tcgtgcggcgagttt |  |  |  |  |  |  |
| 57 | PSA311 | agcattacaatcggcttcacc | 159 | | | *avrPto5* | qRT-PCR | This study |
|  | PSA312 | gcatatcgctccaatcagtcag |  |  |  |  |  |  |
| 58 | PSA313 | acttgacgggaaccgactatga | 224 | | | *hopAZ1* | qRT-PCR | This study |
|  | PSA314 | cgattctgatggcttgacga |  |  |  |  |  |  |
| 59 | PSA315 | ggccgagaagcctgaaggt | 240 | | | *hopAE1* | qRT-PCR | This study |
|  | PSA316 | tccgcaagggagcgtagat |  |  |  |  |  |  |
| 60 | PSA317 | gctccttagcggatggc | 150 | | | *hopH1* | qRT-PCR | This study |
|  | PSA318 | gcagaggcagaccgtgtt |  |  |  |  |  |  |
| 61 | PSA319 | ctgcgctggcgtatgga | 277 | | | *hopZ5* | qRT-PCR | This study |
|  | PSA320 | ccgctgactgtctcggacttat |  |  |  |  |  |  |
| 62 | PSA321 | gaggctgacctgttgatgtatg | 284 | | | *hopZ3* | qRT-PCR | This study |
|  | PSA322 | cccttgtctttagtaacggttgtat |  |  |  |  |  |  |
| 63 | PSA323 | cgtgttctacgacgccttca | 195 | | | *hopAU1* | qRT-PCR | This study |
|  | PSA324 | cccgtctcgctgatttcg |  |  |  |  |  |  |
| 64 | PSA325 | tgctgccactcaagtacgcc | 223 | | | *hrcC* | qRT-PCR | This study |
|  | PSA326 | ccattggagcccaggttgt |  |  |  |  |  |  |
| 65 | PSA327 | ccatcggctttgctcacttcg | 156 | | | *hrcN* | qRT-PCR | This study |
|  | PSA328 | gctgaatcaatggaaagacacccac |  |  |  |  |  |  |
| 66 | PSA329 | tactcatggtggctgtgctcctc | 184 | | | *hrcQa* | qRT-PCR | This study |
|  | PSA330 | tgacgcttgaacaactcacttcg |  |  |  |  |  |  |
| 67 | PSA331 | attgctgccgacccacatc | 184 | | | *hrpK1* | qRT-PCR | This study |
|  | PSA332 | acatacccgcttcgtctacctg |  |  |  |  |  |  |
| 68 | PSA333 | tgctcagggcgtttatcca | 130 | | | *hrpL* | qRT-PCR | This study |
|  | PSA334 | agccaggtctgcggtttact |  |  |  |  |  |  |
| 69 | PSA335 | cgcttcgtactcggcttccc | 213 | | | *hrpR* | qRT-PCR | This study |
|  | PSA336 | cgctgcaactcccagttctttc |  |  |  |  |  |  |
| 70 | PSA337 | cggcacgggcaaagaca | 269 | | | *hrpS* | qRT-PCR | This study |
|  | PSA338 | gcgctcgggtttccaaca |  |  |  |  |  |  |
| 71 | PSA339 | aagtgggcagctcgatgaaa | 204 | | | *hrpZ* | qRT-PCR | This study |
|  | PSA340 | ccgacacccgaaccagaac |  |  |  |  |  |  |
| 72 | PSA341 | gggatcgccttgcacataga | 117 | | | *hrpV* | qRT-PCR | This study |
|  | PSA342 | tacgtccagaaacaggaagtagtga |  |  |  |  |  |  |

**a.** Product size of strains clustered in clade 1, 2, and 3 listed in three columns from left to right, respectively.

**b.** Primers used for clustering *Pseudomonas syringae* pv. *actinidiae* (Psa) biovar 3 strains.

**c.** MLSA, multi-locus sequence analysis; T6SS, type VI secretion system; T3E, type III effector.
