# Supplementary material for: Comparative genomics reveal pathogenicity‐related loci in Pseudomonas syringae pv. actinidiae biovar 3
Source: Mol Plant Pathol. 2019 Apr 26;20(7):923–42. doi: 10.1111/mpp.12803 (PMC6589868; doi:10.1111/mpp.12803)
Supplement: Supplementary file 8 — Table S3 SNPs across the whole genomes of three Psa3 clade 2 strains. [file MPP-20-923-s008.docx]

**Table S3** The SNPs across the whole genomes of three Psa3 clade 2 strains

| No. | ICMP 18884 (genome-plasmid) | | M227-M228-M401 | |
| --- | --- | --- | --- | --- |
| 1 | 1193982 | T | t－t－c | M401-specific |
| 2 | 1194020 | C | c－c－t |  |
| 3 | 1637776 | G | a－a－g |  |
| 4 | 1775918 | C | a－a－c |  |
| 5 | 4322592 | C | c－c－t |  |
| 6 | 4433497 | G | g－g－a |  |
| 7 | 4490153 | G | a－a－g |  |
| 8 | 4814023 | G | g－g－c |  |
| 9 | 6178376 | G | g－g－a |  |
| 10 | 168586 | G | g－a－g | M228-specific |
| 11 | 479306 | C | c－t－c |  |
| 12 | 1533167 | G | g－a－g |  |
| 13 | 2086096 | G | g－a－g |  |
| 14 | 3935052 | T | t－g－t |  |
| 15 | 4523911 | T | t－c－t |  |
| 16 | 5717162 | G | g－a－g |  |
| 17 | 6432908 | G | g－a－g |  |
| 18 | 473809 | A | g－a－a | M227-specific |
| 19 | 1428770 | G | a－g－g |  |
| 20 | 4699686 | G | a－g－g |  |
