# Supplementary material for: Comparative genomics reveal pathogenicity‐related loci in Pseudomonas syringae pv. actinidiae biovar 3
Source: Mol Plant Pathol. 2019 Apr 26;20(7):923–42. doi: 10.1111/mpp.12803 (PMC6589868; doi:10.1111/mpp.12803)
Supplement: Supplementary file 9 — Table S4 Summary of the results of the comparative secretome analysis. [file MPP-20-923-s009.docx]

**Table S4** Summary of the results of the comparative secretome analysis

|  | Number of proteins identified (unique peptide = 1) | | |  | Number of proteins identified (unique peptides ≧ 2) | | |
| --- | --- | --- | --- | --- | --- | --- | --- |
|  | Total | T3SS^a^ | T3Es^b^ |  | Total | T3SS | T3Es |
| Number of proteins differentially secreted (*P*<0.01) | 13 | 0 | **2** |  | 61 | **4** | **11** |
| Number of proteins: M227/M228 >1.5 | 5 | 0 | 0 |  | 12 | 0 | 0 |
| Number of proteins: M227/M228 = 1-1.5) | 0 | 0 | 0 |  | 9 | 0 | 0 |
| Number of proteins: M228/M227 > 1.5 | 4 | 0 | **2** |  | 22 | **4** | **11** |
| Number of proteins: M228/M227 = 1-1.5 | 4 | 0 | 0 |  | 18 | 0 | 0 |

**a.** T3SS indicates type III secretion system components, helper protein and the discontinued effectors.

**b.** T3Es indicates type III effectors.
